# The Developmental Enhancement of a C_4_ System With Non-Typical C_4_ Physiological Characteristics in *Salsola ferganica* (Kranz Anatomy), an Annual Desert Halophyte

**DOI:** 10.3389/fpls.2020.00152

**Published:** 2020-03-06

**Authors:** Yanxia Liu, Tayier Maimaitijiang, Jinghua Zhang, Yali Ma, Haiyan Lan

**Affiliations:** Xinjiang Key Laboratory of Biological Resources and Genetic Engineering, College of Life Science and Technology, Xinjiang University, Urumqi, China

**Keywords:** C_4_ photosynthetic system, developmental enhancement, Kranz-type anatomy, *Salsola ferganica*, *Suaeda aralocaspica*

## Abstract

Variations of photosynthetic structures in different tissues or cells are in coordination with changes in various aspects, e.g. physiology, biochemistry, gene expression, etc. Most C_4_ plant species undergo developmental enhancement of the photosynthetic system, which may present different modes of changes between anatomy and physiology/biochemistry. In the current study, we investigated a Kranz-type C_4_ species *Salsola ferganica* with the progressive development of photosynthetic (PS) structure, performance of PS physiology, induction of PS enzymes, and transcriptional and translational regulation of PS genes, results revealed that *S. ferganica* presented C_3_ type anatomy in cotyledons but C_4_ type in leaves (C_3_/L_4_), with the C_4_ system separation of initial carbon fixation in the palisade mesophyll (M) cells and the following incorporation into triosephosphates and sugars in the bundle sheath (BS) cells, respectively. The BS cells continuously surrounded the vascular bundles and water storage cells in leaf anatomic structure. Compared to the single-cell C_4_ species *Suaeda aralocaspica*, *S. ferganica* exhibited similar developmental enhancement of C_4_ syndrome temporally and spatially in anatomic structures, enzyme activities, and gene expression, which suggests that completion of differentiation of the photosynthetic system is necessary for a C_4_ assimilation pathway. Besides, *S. ferganica* also displayed some different characteristics compared to *S. aralocaspica* in photosynthetic physiology, e.g. a more flexible δ^13^C value, much lower phosphoenolpyruvate carboxylase (PEPC) activity, and an insensitive response to stimuli, etc., which were not typical C_4_ characteristics. We speculate that this may suggest a different status of these two species in the evolutionary process of the photosynthesis pathway. Our findings will contribute to further understanding of the diversity of photosynthesis systems in Kranz-type C_4_ species and the *Salsola* genus.

## Introduction

Photosynthetic carbon assimilation pathways at least include C_3_, C_4_, CAM (Crassulacean acid metabolism), and/or C_2_ (C_3_-C_4_ intermediate) types (Raghavendra and Das, 1993; Khoshravesh et al., 2016), each is associated with distinct features of leaf anatomy, physiology, biochemistry, etc. (Sage and Stata, 2015). Compared to the C_3_ type, C_4_ photosynthesis is advantageous when limited carbon acquisition occurs under high temperature, drought, or saline conditions (Sage, 2004). According to documentation, Amaranthaceae (Chenopodiaceae has been classified into this family currently) possesses the most diverse photosynthetic types and the largest number of C_4_ species in dicotyledonous plants (Pyankov et al., 2000; Pyankov et al., 2001; Kadereit et al., 2003). As one of the largest genera in the family, *Salsola* has the most abundant photosynthetic types recorded so far (Pyankov et al., 2010; Wen and Zhang, 2011), in which many species are widely distributed and can adapt to extreme desert conditions (Wei et al., 2008; Ma et al., 2016). The classical C_4_ photosynthetic pathway differentiates a specialized leaf anatomy (Kranz structure), which consists of two distinct photosynthetic cell types (mesophyll [M] cells and bundle sheath [BS] cells) (Hatch, 1987); whereas single-cell (SC) C_4_ plants can accomplish C_4_ and C_3_ cycles within the same chlorenchyma cell by biochemical compartmentation of the related enzymes and separation of dimorphic chloroplasts in distinct positions (Erlinghaeuser et al., 2016). So far, four SC-C_4_ terrestrial species have been recorded, i.e. *Bienertia sinuspersici*, *Bienertia Cycloptera*, *Bienertia kavirense*, and *Suaeda aralocaspica*, all belonging to the Amaranthaceae (Chenopodiaceae before), which have different spatial distributions of organelles and photosynthetic enzymes in a single chlorenchyma cell (Lung et al., 2012). Usually, behaviors (distribution, activity, etc.) of photosynthetic enzymes in single cells will be correspondingly changed with the structural adjustment (Voznesenskaya et al., 2001a). These variations of photosynthetic anatomy and enzyme function may promote a deeper understanding of carbon assimilation pathway in plants.

Studies have shown that similar anatomy may express different physiological and biochemical characteristics, e.g. plants with C_4_ structure may act with C_3_-like behavior (Edwards et al., 1990; Wen and Zhang, 2015): it has been suggested that these species might be in the process of evolution from a C_3_ to C_4_ photosynthetic pathway on both anatomical and biochemical levels (Ku et al., 1983). Four C_3_-C_4_ intermediate species, *Flaveria anomala*, *Flaveria liaris*, *Flaveria pubescens*, and *Flaveria ramosissima* in the *Flaveria* genus have Kranz-like leaf anatomy, whereas the activities of C_4_ enzyme phosphoenolpyruvate carboxylase (PEPC), pyruvate orthophosphate dikinase (PPDK), etc. and some photosynthetic physiological characteristics are similar to C_3_ species (Ku et al., 1983). In the *Salsola* genus, *Salsola arbusculiformis* has the intermediate anatomic features of two-to-three layers of M cells and Kranz-like BS cells, while the photosynthetic enzyme activity, immunolocalization, and ^14^CO_2_ labeling of initial fixation products are presented as a C_4_ type (Voznesenskaya et al., 2001b); a C_4_ anatomic structure in *Salsola laricifolia*, however, appears with C_3_ or C_3_-C_4_ physiological characteristics (Wen and Zhang, 2015). The inconsistency between photosynthetic biochemical characteristics and anatomic structures in *Salsola* species cannot include all the cases (Voznesenskaya et al., 2013). All available evidence suggests that the evolutionary process from C_3_ ancestors to C_4_ plants occurred many times independently in taxonomically diverse groups (Ku et al., 1983; Soros and Dengler, 2001; Voznesenskaya et al., 2013), which may result in various intermediates matched or not matched in between anatomy and physiological or biochemical characteristics.

All forms of C_4_ species studied so far have been observed with the gradient differentiation of the photosynthetic structure and biochemistry, which finally results in establishment of a fully functional C_4_ syndrome (Koteyeva et al., 2014; Koteyeva et al., 2016). In the Kranz-type C_4_ species representatives, maize and *Arundinella hirta* in the Poaceae, the latter has not only M and BS cells (normal Kranz cells) but also another type of Kranz cells (named as distinctive [D] cells), which are not associated with vascular bundles (VB); however, all cell types show progressive differentiation and enzyme induction pattern, i.e. PEPC or ribulose-1,5-bisphosphate carboxylase (RUBPC) accumulates in M, BS, or D cells along the base-to-tip maturation gradient in developing leaves (Langdale et al., 1988; Wakayama et al., 2003). *Suaeda taxifolia* and *Suaeda eltonica* (Chenopodiaceae), with M and BS cells distributed around the leaf periphery and surrounding VB in the central plane, respectively, both present a longitudinal gradient development of photosynthetic structure and biochemistry in young leaves. In coordination with formation of Kranz anatomy, the chloroplast dimorphism and mitochondrial differentiation are established, and the expression of PPDK, NAD-malic enzyme (NAD-ME), RUBPC, or PEPC is significantly increased in parallel (Koteyeva et al., 2011a). Different types of anatomy between *Cleome angustifolia* and *Cleome gynandra* (with single and multiple Kranz units, respectively) suggest the independent evolutionary origins of C_4_ photosynthesis, but they present similar developmental enhancement of C_4_ system (Koteyeva et al., 2014). In SC-C_4_ species *S. aralocaspica* and *B. sinuspersici*, a gradual enhancement of development along a longitudinal gradient of the young leaf is accompanied by increasing activity of photosynthetic enzymes (e.g. PEPC and RUBPC) (Voznesenskaya et al., 2003; Lara et al., 2008; Koteyeva et al., 2016). Taken together, the progressive development of C_4_ anatomy and related biochemistry should be an adaptive strategy for C_4_ species formed in the long-term evolutionary process.

In the *Salsola* genus, there are a total of nine anatomic types among the C_4_ plant species (Wen and Zhang, 2011), which makes this genus a good candidate for the study of photosynthetic related anatomy, physiology, and biochemistry (Voznesenskaya et al., 2013). *Salsola ferganica* is an annual herbaceous halophyte of *Salsola* and distributed in desert areas of high temperature, drought, and salinization (Zhao et al., 2002; Ma et al., 2018). So far, reports on photosynthetic structures in *S. ferganica* were limited (Wen et al., 2010), and the relevant physiology, biochemistry, or gene expression on photosynthesis has not been documented. Our previous work on *S. ferganica* revealed that the photosynthetic structure and δ^13^C value significantly varied with developmental progression and environmental changes. Based on our observations and updates on the *Salsola* genus, and by employing SC-C_4_ plant species *S. aralocaspica* as the control, in the present study, we address the following questions: (1) Does the photosynthetic anatomy in *S. ferganica* become optimized with plant development progression and if so how does it change? (2) How do the physiology and biochemistry processes progress in coordination with photosynthetic structure differentiation? (3) What photosynthetic enzymes and genes act in the process? By addressing these questions, our findings expand our knowledge of the phenomenon of developmental enhancement of the C_4_ system in *S. ferganica* and increase our understanding of the physiology and biochemistry of carbon fixation in photosynthetic pathways.

## Materials and Methods

### Plant Cultivation and Treatments

*S. ferganica* and *S. aralocaspica* seeds were sown in pots containing perlite: vermiculite (1:3, v/v) in a greenhouse. After emergence, seedlings were cultivated at 18–37°C (Outdoor: 15–36°C), 5–30% relative humidity, 50–300 (Outdoor: 400–2,000) μmol m^-2^ s^-1^ light source, and a 14–16 h light/8–10 h dark photoperiod. Half-strength Hoagland solution (Hoagland and Arnon, 1950) was applied at 2-week intervals. *Chenopodium glaucum* (Chenopodiaceae) and *Nicotiana tabacum* were grown under the same cultivation system for assay of PEPC and RUBPC activity.

For paraffin section preparation (for cotyledon or leaf anatomic structure, starch staining, immuno-histochemical localization of photosynthetic enzyme assays), samples were collected as described in **Supplementary Figure 1**. The image was taken from an indoor plant (the morphology was different from that arising under natural field conditions as shown in **Supplementary Figure 2**). Three types of leaves from different parts of the plant were collected according to the following criteria: 0.5–0.6 cm leaf on the top part, 1.3–1.5 cm (*S. ferganica*) or 1.8–1.9 cm (*S. aralocaspica*) on the upper part, 1.0 cm (*S. ferganica*) or 2.0 cm (*S. aralocaspica*) on the lower part, respectively; each of the different sizes of leaves were carefully divided into tip, middle, and bottom segments, which were then fixed in formalin acetic alcohol (FAA) fixation solution. For qRT-PCR or western blotting analysis of photosynthetic gene or enzyme expression, different leaf segments were immediately frozen in liquid nitrogen for RNA or protein extraction.

For NaCl treatment, different concentrations of NaCl solution (100, 300, 500 mmol L^-1^) prepared with half-strength Hoagland solution were applied to pots from seed sowing time at an interval of 5 days till the experiment was finished: each time the soil matrix was thoroughly saturated with appropriate NaCl solutions. Half strength Hoagland solution was used as the control medium. Four samples of each treatment at 7^th^ d, 15^th^ d, and 30^th^ d after seedling emergence were collected, and ground on ice upon being frozen in liquid nitrogen for immediate assay or stored at −80°C for a short time.

### Observation of Micro-Structure of Leaf, Starch Staining or Immuno-Histochemical Localization

The first completely developed leaf from the top of plant was sampled and three leaves for each species were collected, each leaf was cut into three fragments (tip, middle, bottom) (Koteyeva et al., 2016). FAA solution (90 ml 50% ethanol: 5 ml formaldehyde: 5 ml glacial acetic acid) was used to fix plant tissues. In order to immediately and thoroughly fix the materials, the sealed small glass bottle containing FAA solution and tissues was placed under vacuum repeatedly and gently with a syringe to evacuate the air until the tissues fell down to the bottom, the bottles were then placed at 4°C for 24 h. Through a series of ethanol dehydration, xylene clearing, paraffin inclusion, and embedding, the paraffin blocks containing tissues were sliced into 6–12 μm sections with a microtome (Leica RM2126), and were then expanded and deparaffinized. For micro-structure observation, sections were counterstained with 1% safranine and 1% fast green; for starch staining, after 1 h staining with 1% safranine, slides were immersed into 1% I_2_-KI stock solution (8 g KI + 1 g I_2_ dissolve in 100 ml distilled water) for 20–30 min, then quickly and gently washed with trichloroethane followed by rinsing in distilled water for 30 s. For immuno-histochemical localization, sections were spread on siliconized slides, and treated with 3% hydrogen peroxide methanol solution for 10 min to remove endogenous peroxidase and then rinsed with PBST (phosphate buffered saline [0.01 M, pH 7.2–7.4] [Cat. No. P1010, Solarbio, China] containing 0.05% Tween-20) for 5 min (three times). Following microwave heating repair at 95°C in citrate buffer for 11 min, the slides were rinsed with PBST as described above. Then slides were blocked with 1% bovine serum album (BSA) for 30–60 min, incubated with 1:10 diluted (with 1% BSA) primary antibodies of RUBPC and PEPC, which were made by Abmart (Abmart Shanghai Co., Ltd., China). The epitopic amino acid sequences for polyclonal primary antibody of PEPC and RUBPC were CEKLSSIDAQLR (N-terminal phosphorylation site of typical plant type of PEPC) and QARNEGRDLAREGN (conserved amino acids of plant RUBPC large subunit), respectively. After incubation at 37°C for 1 h, slides were rinsed with PBST buffer for 6 times/15 min each, then incubated with horseradish peroxidase (HRP) labeled secondary antibody (1:500 diluted with 1% BSA) at 37°C for 1 h. The treated slides were stained with diaminobenzidine (DAB) (Sangon, Shanghai), and rinsed with PBST for 5 times/15 min each (Huang et al., 2015). For all above, sections were then treated in absolute ethanol for 5 min (two changes) and then in xylene for 5 min (two changes) for dehydration. After mounting, slides were sealed with neutral balsam and incubated at 37°C for drying and finally inspected. The images were acquired by an inverted fluorescence microscope (Nikon ECLIPSE Ti-E, Japan), and photographs were taken by using Nis-Elemens software (Japan).

### Measurement of MC: BSC or C_d_: C_p_ Area Ratio and Chloroplast Size

Leaves from outdoor plants (4–8 weeks old) were used for measurement. The paraffin sections were prepared before inspection under the microscope (as described above). For area of mesophyll cells (MC) and bundle sheath cells (BSC) in *S. ferganica*, or C_d_ and C_p_ (referring to the distal end and proximal end areas of a single chlorenchyma cell, respectively; these were obtained from the division at the apparently chloroplast-free area) in *S. aralocaspica*, the tip segment of mature leaf was employed, 5 leaves with 5 views of each, and 6 MCs and BSCs (*S. ferganica*) or chlorenchyma cell (*S. aralocaspica*) of each view were measured; for the chloroplast size, 3 leaves with 3 cells of each, and 5 chloroplasts from each cell were measured. Two measurements were conducted under 100×oil immersion lens, and Image J 1.48U software (National Institutes of Health, USA) was used in calculation of cell area and chloroplast size; Microsoft Excel software was used in the data analysis.

### Determination of *δ* Value of ^13^C Carbon Isotope

Leaves collected from *S. ferganica* and *S. aralocaspica* (cultivated in greenhouse or outdoors) were immediately placed at 150°C for 30 min to deactivate enzymes, then transferred to an aerated oven at 70°C overnight, finally the tissue was ground into an homogeneous powder and sent for δ^13^C value determination (Beijing Ailemengtuo Science and Technology Co. Ltd., Beijing). δ^13^C value was calculated by reference to the international standard PDB [Pee Dee Belemnite (Bender et al., 1973)].

### Assay of PEPC and RUBPC Activity

Enzyme-catalyzed reactions were employed in determination of activity of PEPC and RUBPC. For PEPC, young fresh leaves (0.1 g) from the upper part of plant were homogenized on ice in extraction buffer (1.0 ml) containing 100 mM Tris-H_2_SO_4_ (pH 8.2), 7 mM β-mercaptoethanol, 1 mM EDTA, and 5% glycerol. The homogenate was centrifuged at 1,800 *g* for 15–20 min at 4°C and the supernatant was immediately used. The following steps were performed according to protocols described by Cao’s reaction mixture (1.0 ml) consisted of 70 mM MgSO_4_·7H_2_O (143 μl), 70 mM NaHCO_3_ (143 μl), 14 mM phosphoenolpyruvic acid (286 μl), 5 mM NADH (429 μl) with only modification by addition of 10 U malate dehydrogenase and 20 μl crude enzyme to initiate reactions (Cao et al., 2015). For RUBPC assays, young fresh leaves (0.1 g) from the upper part of the plant were ground on ice in extraction buffer (1.5 ml) containing 40 mM Tris-HCl (pH 7.6), 10 mM MgCl_2_·6H_2_O, 0.25 mM EDTA, and 5 mM glutathione, followed by centrifugation as mentioned above, the supernatant was then immediately used for assay. The reaction mixture (1.0 ml) consisted of 0.2 mM NaHCO_3_ (67 μl), reaction buffer (467 μl) (100 mM Tris-HCl [pH 7.8], 12 mM MgCl_2_·6H_2_O, 0.4 mM EDTA), crude enzyme extract (133 μl), 5 mM NADH (67 μl), 50 mM ATP (67 μl), 50 mM DL-dithiothreitol (67 μl), distilled water (33 μl), 160 U·ml^-1^ phosphoglycerate kinase (33 μl), 160 U·ml^-1^ phosphoglyceraldehyde dehydrogenase (33 μl), and 25 mM RuBP (33 μl) (Cao et al., 2015). The absorbance of both reaction mixtures was recorded on a UV-3010 spectrophotometer (Shimadzu, Japan) for 3 min at 340 nm. Enzyme activity of PEPC and RUBPC was defined as 0.01 optical density value decrease per minute equaling 1 U (NC-IUB, 1979). Total protein content was determined by measuring the absorbance at 595 nm. For diurnal changes of enzyme activity, the light intensity, temperature, and relative humidity indoor or outdoor were measured simultaneously at 2 h intervals (**Table 1**).

**Table 1:** Light intensity, temperature, and humidity at different time points.

Time points	Indoor time							
	08:00	10:00	12:00	14:00	16:00	18:00	20:00	22:00
Light Int. (μmol·m^-2^·s^-1^)	64.2	160.4	171.2	268.5	198.2	153.4	83.09	3.5
Temp. (°C)	19.4	25.9	30.3	36.1	35.9	34.7	34.6	30.1
Hum. (%)	30	23	18	10	10	10	10	10
**Time points**	**Outdoor time**							
	**08:00**	**10:00**	**12:00**	**14:00**	**16:00**	**18:00**	**20:00**	**22:00**
Light Int. (μmol·m^-2^·s^-1^)	467.9	1143.5	1803.6	1916.9	1596.8	826.9	539.2	10.16
Temp. (°C)	19.3	22.2	27.3	35.8	31.7	29.9	28.6	25.3
Hum. (%)	30	22	10	10	10	10	10	10

Int., intensity; Hum., humidity; Temp., temperature.

### Quantitative RT-PCR (qPCR)

Total RNA was isolated from *S. ferganica* or *S. aralocaspica* seedlings using a Plant RNA Kit (R6827-02, Omega, US). Reverse transcription was performed with 1 µg of total RNA in 20 µl by using M-MLV (TaKaRa, Dalian, China) according to the manufacturer’s instruction. qPCR primers are shown in **Table 2**. For primer design, the coding regions of *PEPC*1, *PEPC*2, *PPDK*, *RBCL* (large subunit of RUBPC) from different species reported in Chenopodiaceae were acquired, and the degenerate primers were designed to get short DNA sequences of each gene from *S. ferganica* or *S. aralocaspica*, respectively; then the accurate qPCR primers were designed based on the acquired short sequences of each gene. For *PEPC*1 and *PEPC*2 primers, it was necessary to first find out the most conserved region of *PEPC*1 or *PEPC*2 among different species reported in Chenopodiaceae, then to select the most different sequences between *PEPC*1 and *PEPC*2 from these conserved regions, which could only amplify one of these two genes in *S. ferganica* or *S. aralocaspica*. *β-ACTIN* from *S. ferganica* or *β-TUBULIN* from *S. aralocaspica* was used for an internal reference gene. The above amplification was performed at the following conditions: 95°C 2 min followed by 40 cycles of 95°C 5 s, 60°C 30 s. qPCR was performed with SuperReal PreMix Plus (SYBR Green) Kit (Cat. FP205-02; Tiangen, China) and ABI 7500 Real time PCR system (Applied Biosystem, USA). Relative quantification of specific mRNA level was calculated using the cycle threshold (Ct) 2^−ΔΔCt^ method (Shi and Chiang, 2005), where ΔΔC_T_ = ΔC_T__target_ _sample_ - ΔC_T__control_ _sample_, ΔC_T__target_ _sample_= C_T__test_ _gene_ - C_T__reference_ _gene_. Three samples (biological replicates) of each treatment were duplicated (technical replicates) in each qPCR experiment. The final value of relative quantification was described as fold change of gene expression in target sample compared to the control of the bottom segment of leaf in each test gene.

**Table 2:** Primer sequences used in the present study.

Plant species	Gene	Primer sequence 5’-3’	
		Forward	Reverse
*S. ferganica*	*PEPC*1	GGCATACACTCTGAAGCGGATAC	GCATTCCAGCAGCAATACCCTT
	*PEPC*2	TGTCCATTTGGCGAGGGG	CCTTTGAGATGTGGGGCCT
	*PPDK*	CATCTTGACCAACTTCTTCATCCAC	ACTCTTTCCTTGTGCTTGCCAG
	*RBCL*	GGCAGCATTCCGAGTAAGTCC	TCAAAAAGGTCTAAGGGGTACGCTA
	*β-ACTIN*	TCTACAATGAGCTTCGTGTGGC	CACCATCACCAGAATCCAGCAC
*S. aralocaspica*	*PEPC*1	GCATCCACCAAGCTCTCCTAAG	CCATACTCCAACTCAGGCG
	*PEPC*2	GAACTCTCCAGCGTTTCACTG	CAATGCAGTTTCTGGTGTGG
	*PPDK*	CTGTCCCAGGAGTCAAACAC	CACTGAACTAACTGCTTCCGA
	*RBCL*	ACGGTCGAGCAGTTTATGAATGTC	GTCTTCACATGTACCCGCAGTAGC
	*β-TUBULIN*	CCTTATTCCATTCCCCAGGCTTC	CATCTGCTCATCAACCTCCTTTGTGC

### Western Blotting Analysis of Photosynthetic Enzymes

Leaf samples (approximately 0.2 g) of different developmental stages, i.e. young leaf (YL, 0.2–0.3 cm), intermediate leaf (IL, 0.5–0.6 cm), mature leaf (1.2–1.5 cm), from different positions (plant upper leaf [PUL], plant middle leaf [PML], plant lower leaf [PLL]), and leaf segments (approximately 0.1 g; intermediate leaf divided into three parts towards the bottom, middle, and tip), were used for extraction of the soluble protein according to the method described by Koteyeva et al. (2011b). The supernatant (protein solution) was mixed with loading buffer [250 mM Tris-HCl, pH 6.8, 10% (w/v) SDS, 50% (v/v) glycerol, 5% (v/v) β -mercaptoethanol, and 0.5% (w/v) bromphenol blue] in 4:1 (v:v) and boiled for 10 min, then used for SDS-PAGE after centrifuged at 10,000 *g* for 10 min at 4°C. Protein concentration was determined with a Bradford protein assay kit (Solarbio, Beijing). Protein samples (10 μg of each) were resolved by 12% (w/v) SDS-PAGE, and transferred to a polyvinylidene fluoride membrane for immunoblotting analysis of the photosynthetic enzymes. All the primary antibodies used in the present study were raised against the predicted optimal epitopic antigens of the conserved amino acid sequences of PEPC, PPDK, NAD-ME, RBCL from *S. aralocaspica*, the amino acid residues of the epitopic antigens of these antibodies and the working dilution were as follows: anti-*Sa*PEPC (EKLSSIDAQLR) IgG (1:500), anti-*Sa*NAD-ME (NGRTGHVNQGNNMY) IgG (1:200), anti-*Sa*PPDK (KLATEKGRAAKPSL) IgG (1:200), and anti-*Sa*RBCL (QARNEGRDLAREGN [large subunit of RUBPC]) IgG (1:500), the epitopic antigens of all these enzymes from *S. aralocaspica* could be used in western blotting detection of *S. ferganica* based on sequence analysis. The secondary antibody—goat anti-rabbit IgG (conjugated horseradish peroxidase) (1:2000)—was used for detection. Bound antibodies were visualized by enhanced chemiluminescent (Biosharp, Beijing), and images were acquired by luminescent image analyzer (FUJIFILM LAS-4000, Japan). The bands were quantified by Image J 1.41 (Wayne Rasband National Institutes of Health, USA) and calculated as relative intensity (RI, %). For RI of each enzyme in YL, IL, PUL, PML, and PLL, the 'PUL' was used while for bottom, middle, and tip of leaf, the ‘Tip’ was used as reference in calculations.

### Statistical Analysis

Data were analyzed and graphs were prepared by Graphad Prism 5.0 for Windows (GraphPad Software, San Diego, CA). One-way or two-way ANOVA was used to compare more than two variables. Significant differences were analyzed by a multiple comparison Tukey test at 0.05, 0.01, or 0.001 significance level.

## Results

### Plant Development and Morphological Characteristics of *S. ferganica*

*S. ferganica* is an annual halophyte distributed in harsh natural habitats. Its seed with winged perianth can germinate on the soil surface in the presence of high salinity. Early seedlings of *S. ferganica* presented with two smooth flat lanceolate cotyledons (**Supplementary Figure 2A**), while the true leaf was succulent and appeared as a long clavate shape, more interestingly, which was covered with white, thick, long, and soft trichomes (**Supplementary Figure 2B**), and these remained on the plant from an early seedling to an early adult stage (**Supplementary Figure 2C**). With further plant development, the trichomes became thinner and shorter on leaves until almost invisible (**Supplementary Figures 2D, E**). We found that the dynamics of trichome growth and decline on plants were corresponding to the development rhythms of *S. ferganica*, i.e. trichomes were thick and long at an early stage while shorter and thinner at a later stage, which suggests that trichomes must be an adaptive structure to protect seedling survival.

### Cotyledon and Leaf Anatomic Structures of *S. ferganica* and *S. aralocaspica*

**Figure 1:**
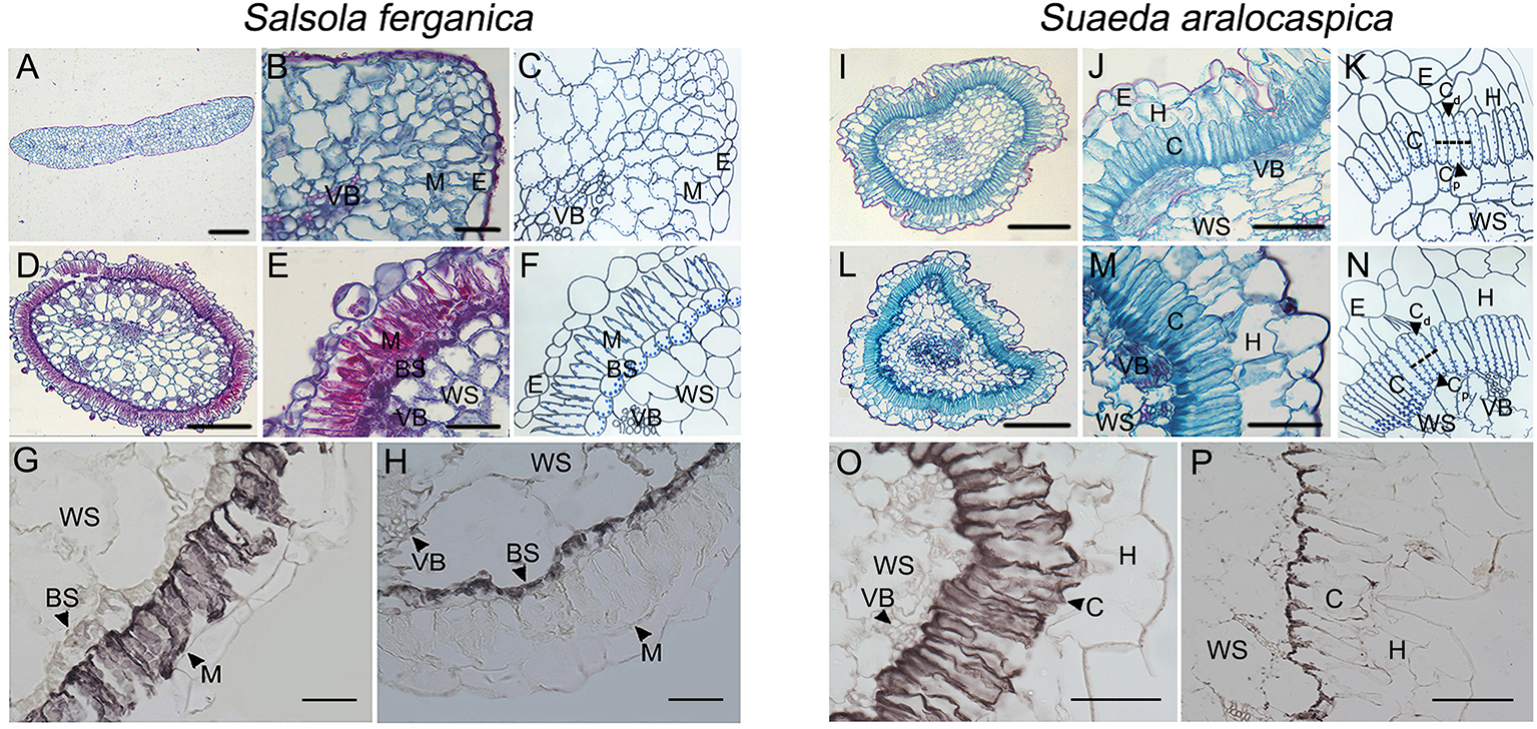
Anatomic structures of cotyledon and leaf and immunohistochemical localization of PEPC and RUBPC of leaf in *S. ferganica* and *S. aralocaspica*. **(A–H)** *S. ferganica*; **(I–P)** *S. aralocaspica*; **(A, D, I, L)** Images of whole leaf cross section; **(B, E, J, M)** Enlarged images of part leaf cross section; **(A, B, I, J)** Cotyledon; **(D, E, L, M)** Leaf; **(C, F, K, N)** The schematic drawing corresponding to B, E, J, M; **(G, O)** PEPC; **(H, P)** RUBPC. BS, bundle sheath; C, chlorenchyma; M, mesophyll; VB, vascular bundle; WS, water storage. The images of anatomic structure and immunohistochemical localization of PEPC and RUBPC were all acquired from mature leaves of outdoor plant. Scale bar in A, D, I, L is 200 μm; B, E, G, H, J, M, O, P is 50 μm.

Besides the different appearance between the cotyledons and the true leaf form in *S. ferganica*, the anatomic structure was also different (**Figures 1A–F**). In the case of the cotyledon, M cells had no significant differentiation in their micro-structure (**Figures 1A–C**); however, M cells of the true leaf were specialized and organized into a layer of elongated, palisade-like cells around a ring of BS cells (Kranz structure) inside (**Figures 1D–F**); in contrast, the appearance and anatomic structures of the cotyledon (**Figures 1I–K**) and leaf (**Figures 1L–N**) of S*. aralocaspica* were similar, in which the chlorenchyma cells were organized as a layer of elongated, palisade-like cells. Analysis of immuno-histochemical localization showed that, in *S. ferganica*, PEPC was distributed throughout the M cells, while RUBPC presented in BS cells (**Figures 1G, H**); in *S. aralocaspica*, PEPC was observed in all of chlorenchyma cells while RUBPC was only found at the proximal end (**Figures 1O, P**).

### Starch Staining in Developing Cotyledon and Leaf in *S. ferganica* and *S. aralocaspica*

**Figure 2:**
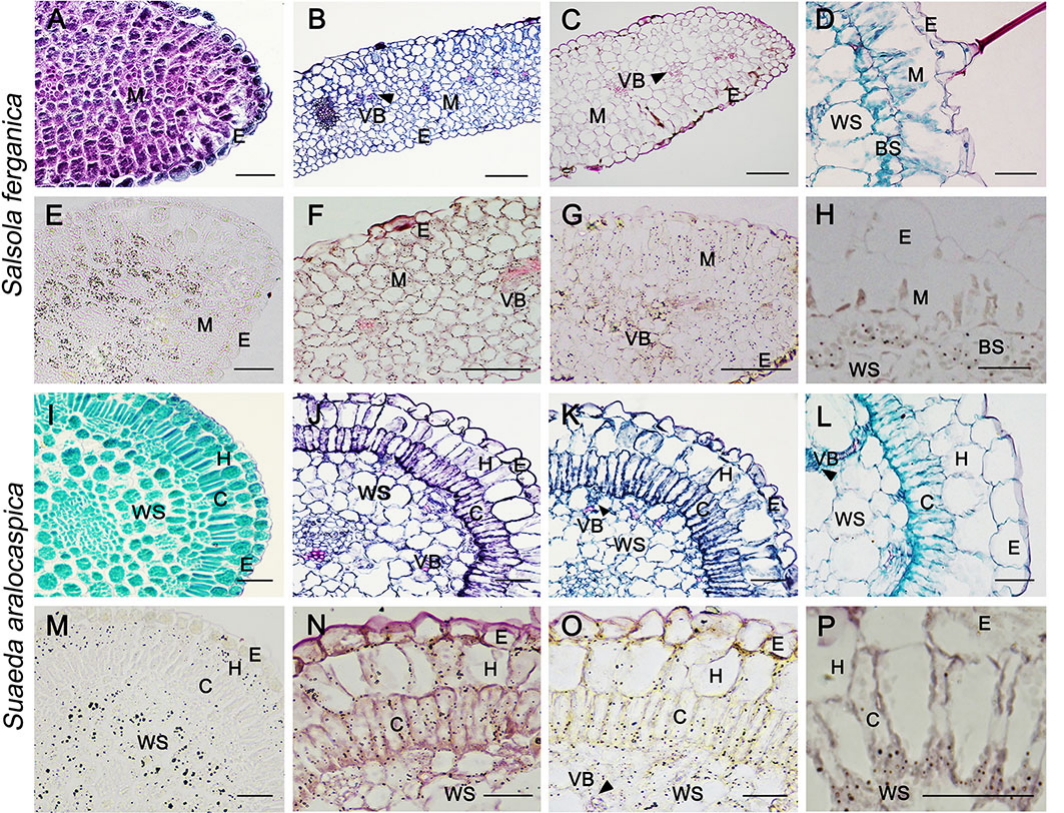
General anatomy (upper panel) and starch localization (lower panel) in developing cotyledon and true leaf of *S. ferganica* and *S. aralocaspica*. **(A, E, I, M)** Cotyledon, 0 d; **(B, F, J, N)** Cotyledon, 4^th^ d; **(C, G, K, O)** Cotyledon, 8^th^ d; **(D, H, L, P)** Mature leaf. BS, bundle sheath; C, chlorenchyma; E, epidermis; H, hypodermis; M, mesophyll; VB, vascular bundle; WS, water storage. Scale bar is 50 μm.

Starch staining in cotyledon at 0 d, 4^th^ d, and 8^th^ d after germination was visualized in both plant species by paraffin sections under the microscope (no true leaf emerged at this moment in either *S. ferganica* or *S. aralocaspica*) (**Figure 2**). Starch granules in the cotyledon of *S. ferganica* (C_3_ structure) at 0 d were mainly accumulated in the interior M cells (**Figures 2A, E**), but were distributed in most of the M cells on the 4^th^ d as the cotyledon developed and the starch staining was stronger in cells near the lower surface than the upper surface (**Figures 2B, F**). At the 8^th^ d, the starch distribution pattern in cotyledons was similar to that of the 4^th^d (**Figures 2C, G**). In the mature true leaf of *S. ferganica* (C_4_ structure), starch granules were mainly distributed in the interior bottom of BS cells, and some in the periplasm of water storage (WS) cells (**Figures 2D, H**).

In *S. aralocaspica*, starch granules in the cotyledon (C_4_ structure) at 0 d were mainly distributed in outer the layer of hypodermal cells and inner WS cells (**Figures 2I, M**). With cell structure differentiation, the single layer of chlorenchyma cells was elongated in the radial direction and formed a palisade-like cell layer with cytoplasm polarized towards the proximal and distal ends. At the 4^th^ d, starch granules were distributed in the peripheral cytosol of chlorenchyma, hypodermal and WS cells (**Figures 2J, N**). At the 8^th^ d, palisade-like chlorenchyma cells became longer, and starch granules were accumulated at the proximal end of chlorenchyma cells (**Figures 2K, O**). In the mature true leaf, starch granules were mainly distributed at the proximal end of apparently elongated chlorenchyma cells (**Figures 2L, P**).

### Characteristics of MC: BSC and C_d_: C_p_ Ratio and δ^13^C Value in *S. ferganica*
and *S. aralocaspica*

**Figure 3:**
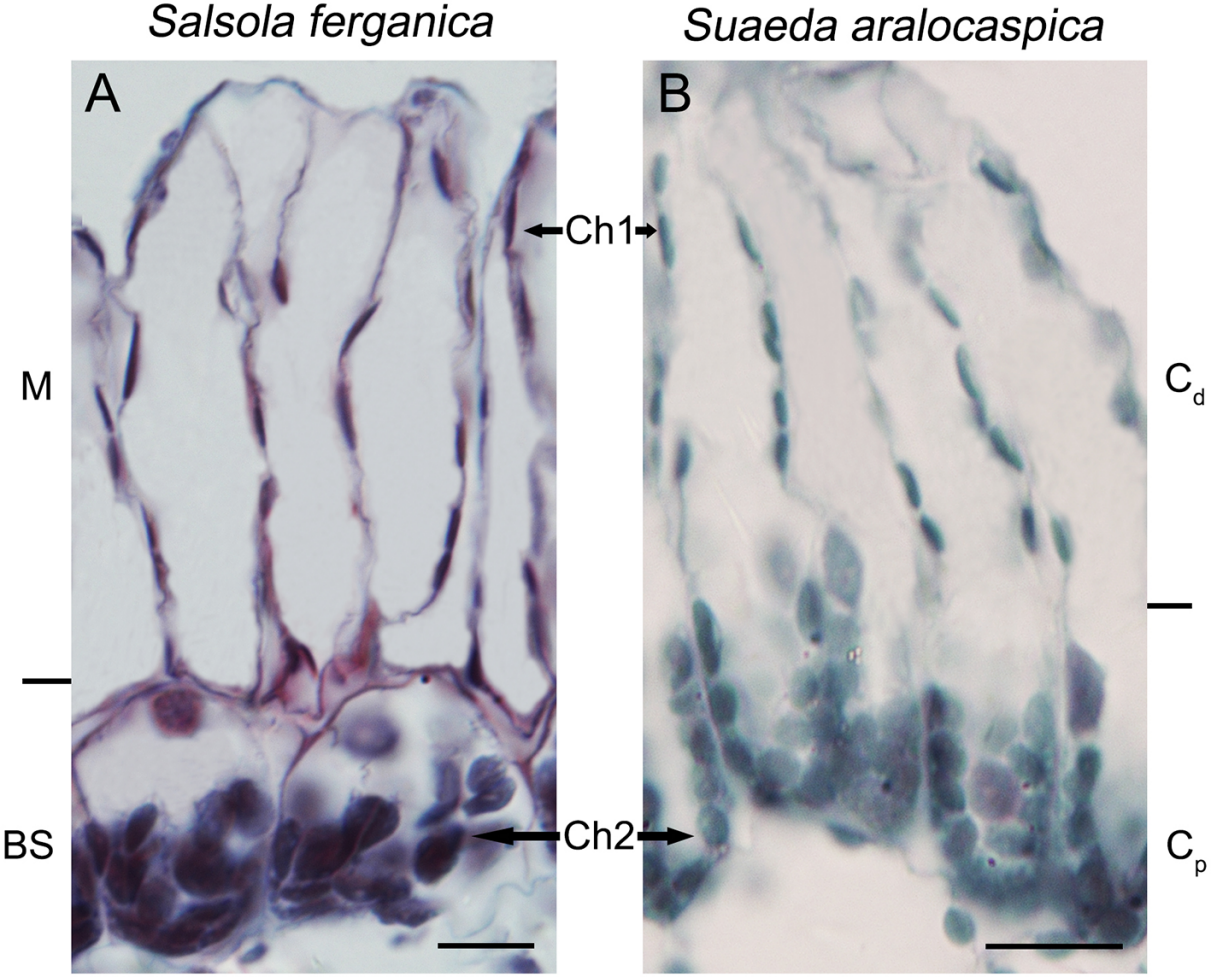
The shapes and sizes of dimorphic chloroplasts in *S. ferganica* and *S. aralocaspica*. **(A)** *S. ferganica*; **(B)** *S. aralocaspica*. Samples were collected from indoor plant. BS, bundle sheath; Ch1, chloroplast in MC (*S. ferganica*) or C_d_ (*S. aralocaspica*); Ch2, chloroplast in BSC (*S. ferganica*) or C_p_ (*S. aralocaspica*); C_d_, distal end of chlorenchyma cell; C_p_, proximal end of chlorenchyma cell; M, mesophyll. Scale bar is 10 μm.

Results of cell area ratio showed that it was 1.42 (MC : BSC) for *S. ferganica*, and 1.67 for *S. aralocaspica* (C_d_ : C_p_ of single chlorenchyma cell). At the same time, two different sizes of chloroplasts were observed in both species, i.e. in the inner BS cell of *S. ferganica* or proximal end of chlorenchyma cells of *S. aralocaspica* oval chloroplasts were observed, but in peripheral M cells or the distal end, long thin oval chloroplasts were present (**Figure 3**; **Table 3**). Analysis of the δ^13^C value indicated that it changed significantly under different conditions in *S. ferganica*, i.e. −16.15‰ in outdoor adult plant in summer; −20.41‰ or −21.73‰ in greenhouse seedlings in winter or greenhouse adult plant in summer. Compared to SC-C_4_ *S. aralocaspica* with values of −14.87‰ in greenhouse seedlings in winter, and the C_3_ species *C. album* (−34.15‰) or *N. tabacum* (−33.10‰) in greenhouse adult plant in summer, the above results suggest that the δ^13^C value of *S. ferganica* is flexible and can adjust with the development progression of the plant and when environmental conditions change (**Table 4**).

**Table 3:** Properties of anatomic structures of mesophyll cells (MC), bundle sheath cells (BSC), chlorenchyma cells (CC), and dimorphic chloroplasts in *S. ferganica* and *S. aralocaspica*.

Species	Cell area ratio^δ^	Dimorphic chloroplast		
		Shape	Size (μm)	Location
*S. ferganica*	1.42 ± 0.29	Oval Long thinoval	5.32 ± 0.87×3.43 ± 1.09 5.41 ± 1.33×1.06 ± 0.24	Inner BSC Peripheral M
*S. aralocaspica*	1.67 ± 0.26	Oval Long thin oval	3.29 ± 0.66×2.63 ± 0.34 4.44 ± 0.84×1.21 ± 0.24	Proximal end of CC Distal end of CC

^δ^, for S. ferganica is MC : BSC, for S. aralocaspica is C_d_ : C_p_ (ratio between the distal end area and proximal end area of the chlorenchyma cell). All data were collected from indoor plants. Values are means ± SE of 15 replicates.

**Table 4:** δ^13^C value of *S. ferganica* and other plant species.

Plant species	Growth condition	Measurement date	Developmental stage	δ^13^C (‰)
*S. aralocaspica*	Greenhouse	Dec, 2015	Early seedling (1 W)	−14.87 ± 0.366
*S. ferganica*	Greenhouse	Dec, 2015	Early seedling (1 W)	−20.41 ± 0.3129
*S. ferganica*	Greenhouse	May, 2016	Early adult plant (2 M)	−21.73 ± 0.2012
*S. ferganica*	Outdoor	May, 2016	Early adult plant (2 M)	−16.15 ± 0.3463
*C. album*	Greenhouse	May, 2016	Early adult plant (2 M)	−34.15 ± 0.6043
*N. tabacum*	Greenhouse	May, 2016	Early adult plant (2 M)	−33.10 ± 0.3284

W, week; M, month. δ^13^C, carbon isotope value. δ^13^C value of C_4_ species is −10‰–−15‰; C_3_ species is −21‰–−30‰ (Sage et al., 1999). Values are means ± SE of three replicates.

### Characteristics of PEPC and RUBPC Activity in *S. ferganica* and *S. aralocaspica*

#### Diurnal Changes

**Figure 4:**
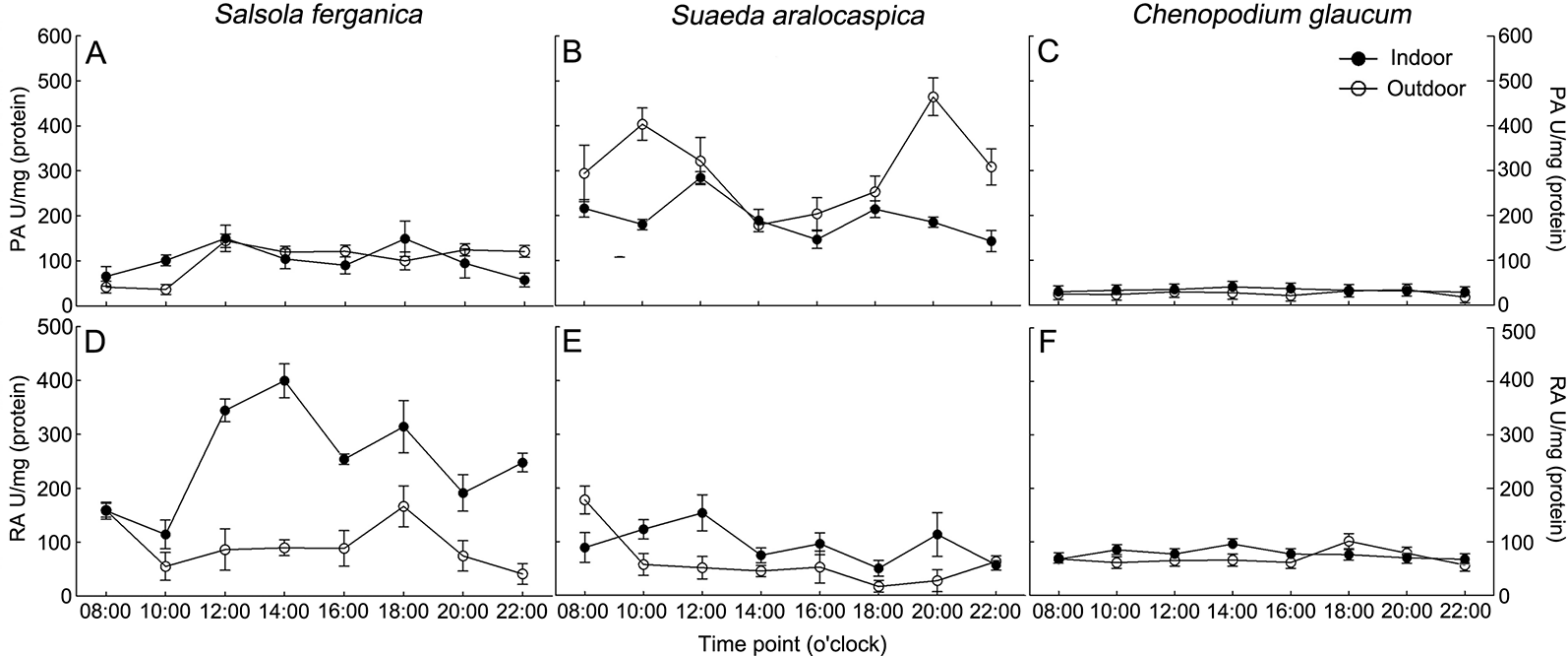
Diurnal changes of PEPC and RUBPC activity in *S. ferganica*, *S. aralocaspica* and *C. glaucum*. **(A, D)** *S. ferganica*; **(B, E)** *S. aralocaspica*; **(C, F)** *C. glaucum*; **(A–C)** PEPC activity; **(D–F)** RUBPC activity. Indoor, in greenhouse; Outdoor, out of greenhouse. PA, PEPC activity; RA, RUBPC activity.

To understand PEPC and RUBPC activity in response to the alteration of light intensity during a whole day, we measured the enzyme activity from 8:00 am (08:00) in the morning until 10:00 pm (22:00) in the evening at 2 h intervals (**Figures 4A–F**). The overall PEPC activity (PA) of *S. ferganica* was much lower than that of *S. aralocaspica* (**Figures 4A, B**), especially that of outdoor plants; while RUBPC activity (RA) exhibited the opposite trend between the two species (**Figures 4D, E**), with higher RA in indoor plants compared to activity in outdoor plants. Between the two enzymes, RA was higher than that of PA in *S. ferganica*, especially from indoor plants (**Figures 4A, D**); while in *S. aralocaspica*, the two enzymes showed the opposite trend (**Figures 4B, E**). The highest PA was observed in outdoor grown *S. aralocaspica* (**Figures 4A, B**), while the highest RA appeared in *S. ferganica* that had been grown indoors (**Figures 4D, E**). Our results indicate a tendency of PA in *S. ferganica* and *S. aralocaspica* to present as a “double peak” pattern; for indoor plants, two higher PA values presented at 12:00 pm (noon) and 6:00 pm (18:00) in the early evening (**Figures 4A, B**); for outdoor plants, these were at 10:00 am or 12:00 pm in the morning and at noon, and at 8:00 pm (20:00) in the evening. As a C_3_ plant control, *C. glaucum* presented a different pattern on PA and RA in response to diurnal changes, which were at a lower activity level and appeared to be insensitive to light intensity (**Figures 4C, F**). Our results suggest that the differences in activity of two photosynthetic enzymes exist between *S. ferganica* and *S. aralocaspica*, in which the former appeared as non-typical C_4_ plant characteristics compared to that of *S. aralocaspica*.

#### Developmental Changes and Salt Responses

**Figure 5:**
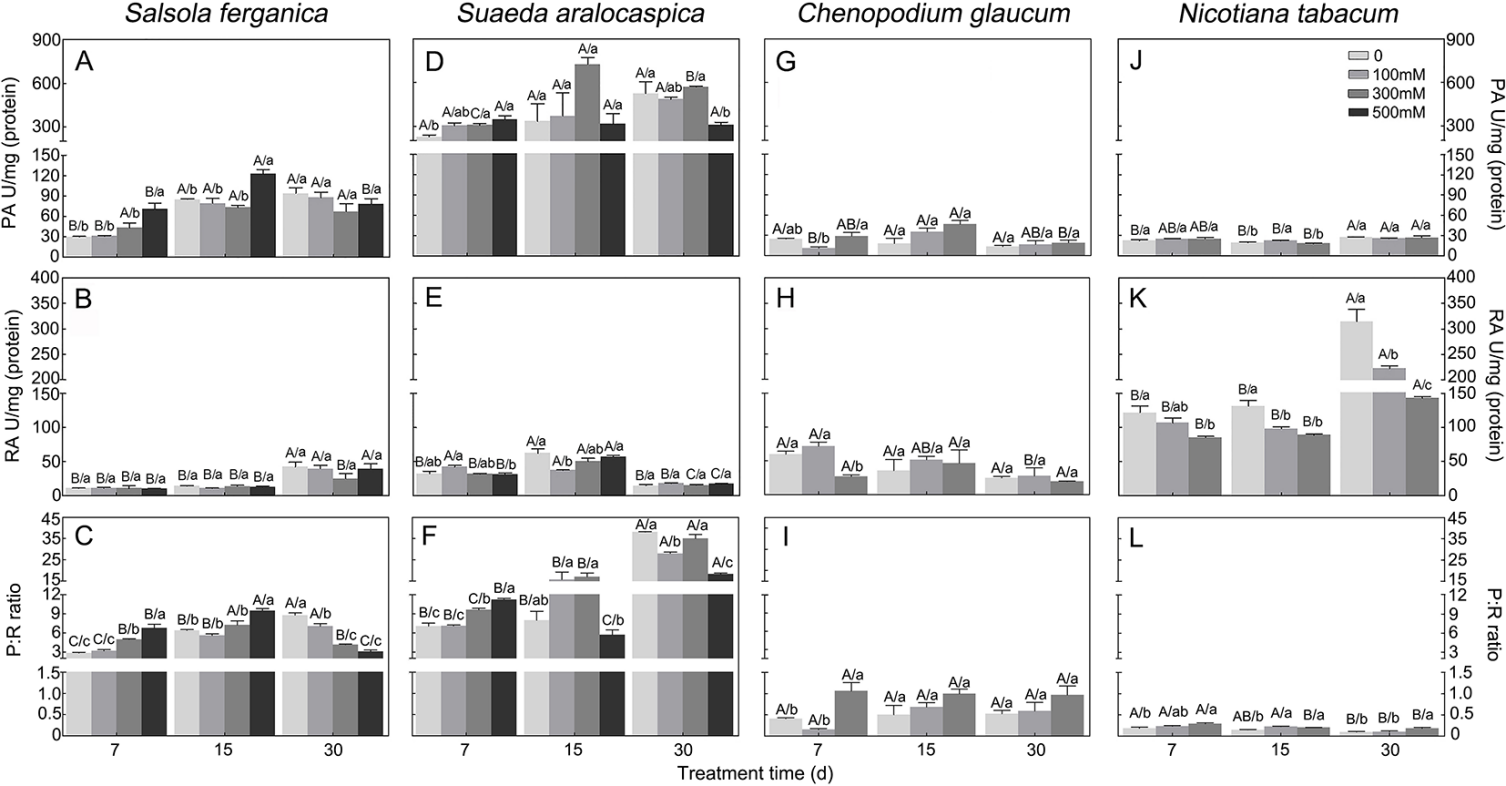
Changes of PEPC and RUBPC activity and its ratio in *S. ferganica*, *S. aralocaspica*, *C. glaucum*, and *N. tabacum* under NaCl treatment at different developmental stages. **(A–C)** *S. ferganica*; **(D–F)** *S. aralocaspica*; **(G–I)** *C. glaucum*; **(J–L)** *N*. *tabacum*; **(A, D, G, J)** PEPC activity; **(B, E, H, K)** RUBPC activity; **(C, F, I, L)** Ratio of PEPC: RUBPC. PA, PEPC activity; RA, RUBPC activity; P: R, PEPC activity: RUBPC activity; 7, 15, 30: Days of seedling after emergence. Different lowercase letters above columns indicate significant differences (*P* < 0.05 or 0.01) among different NaCl concentrations at the same developmental stage; different uppercase letters above columns indicate significant difference (*P* < 0.05 or 0.01) among different developmental stages at the same NaCl concentration. Values are means ± SE of four replicates.

PA and RA under NaCl treatment or different growth periods showed that, for *S. ferganica*, PA was increased at 15 d and 30 d compared to that of 7 d plants, while there was no large change between 15 d and 30 d plants (**Figure 5A**). Moreover, at early developmental time (7 d or 15 d), PA was increased with rising NaCl concentration; whereas RA was increased significantly at 30 d compared to that of 7 d or 15 d; however, there was no large change of PA or RA between 7 d and 15 d plants, or among different NaCl concentrations (**Figure 5B**). For *S. aralocaspica*, PA was increased with seedling development at lower or medium NaCl concentrations between 7 d and 15 d (or 30 d) after emergence (**Figure 5D**); RA was increased at 15 d while reduced at 30 d but there was no significant change among different NaCl concentrations (**Figure 5E**). For the C_3_ species control plants: *C. glaucum* (halophyte) or *N. tabacum* (glycophyte) presented a much lower PA level than that of *S. ferganica* or *S. aralocaspica*, there was almost no large change among different developmental periods or NaCl concentrations (**Figures 5G, J**); RA of *C. glaucum* was at a similar level and pattern to RA in *S. aralocaspica*, and declined with increasing NaCl concentration (**Figure 5H**); while RA of *N. tabacum* was the highest among the four species, and was increased with seedling development, although reduced with increasing NaCl concentration (**Figure 5K**). The above changes of PA and RA resulted in an increase of PEPC : RUBPC ratio (P:R) from 7 d to 15 d but this decreased at 30 d plants in *S. ferganica*: the highest value observed was about 10 (500 mM NaCl at 15 d) and the lowest was around 3 (control at 7 d and 500 mM NaCl at 30 d) (**Figure 5C**). For *S. aralocaspica*, the P:R ratio was increased from 7 d to 30 d except for the higher NaCl concentrations at 15 d or 30 d (**Figure 5F**), the highest value was about 40 (control at 30 d) and the lowest was around 5 (500 mM NaCl at 15 d); for *C. glaucum*, P:R was increased with increasing NaCl concentration and most of the values were lower than 1.0 (**Figure 5I**); for *N. tabacum*, P:R was reduced with seedling development and in general lower than 0.5 (**Figure 5L**). Results of PA and P:R suggest that the photosynthetic physiology of *S. ferganica* presents a difference compared to that of *S. aralocaspica*, which exhibited as non-typical C_4_ characteristics.

### Developmental Changes of Amount and Localization of PEPC and RUBPC in Leaf of *S. ferganica* and *S. aralocaspica*

**Figure 6:**
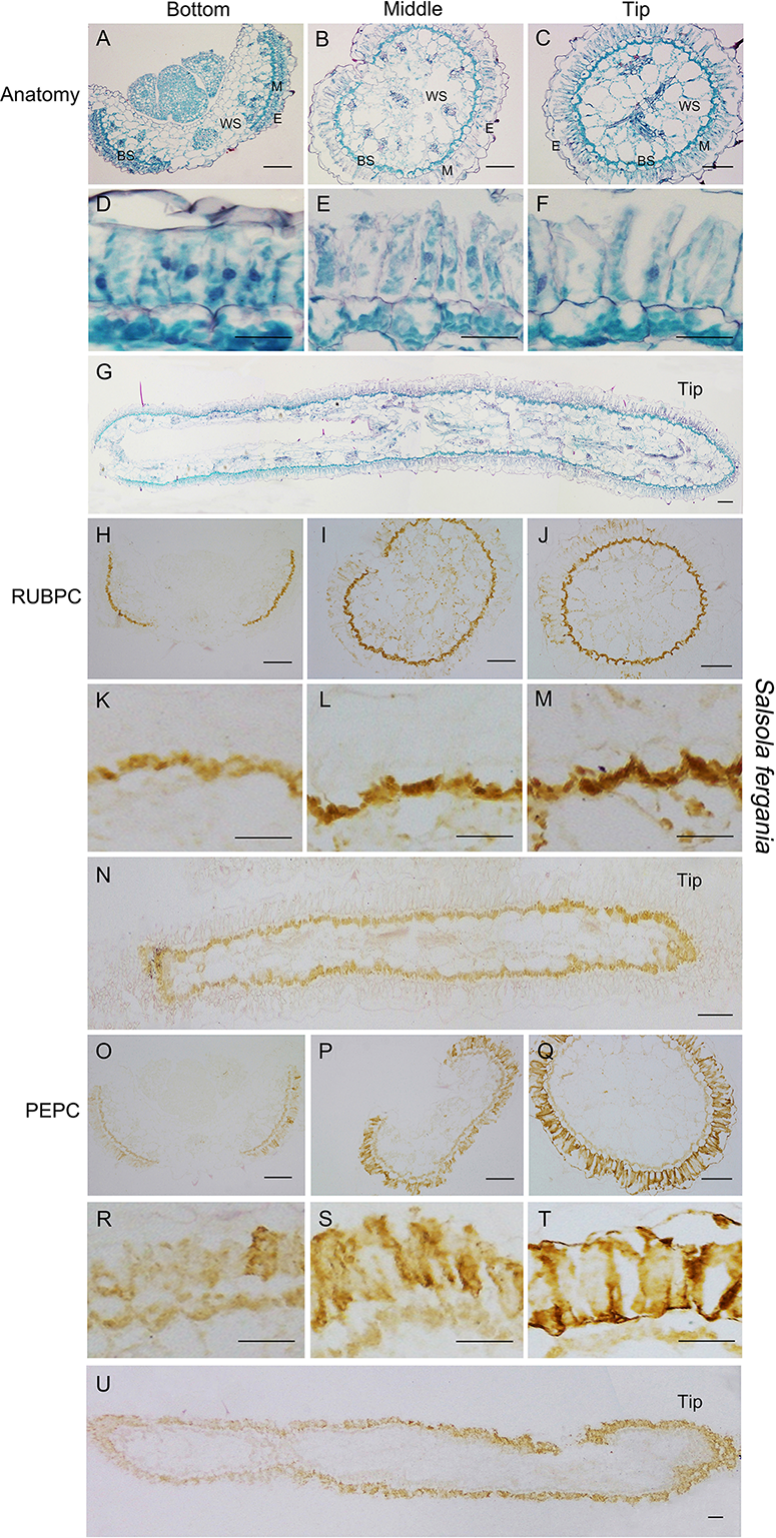
Anatomic structure of leaf and *in situ* immuno-histochemical localization (ISIHL) of RUBPC and PEPC of *S. ferganica* with transverse and longitudinal sections of young leaves (0.5–0.6 cm), showing a progressive development with gradual structure differentiation. **(A–G)** Anatomic structure of young leaf; **(H–U)** ISIHL results; **(A–C)** Transverse sections from the bottom to tip of leaf; **(D–F)** Detailed views corresponding to A–C; **(G)** Longitudinal sections of leaf; **(H–J)** ISIHL of RUBPC (RBCL) at the bottom, middle, and tip of leaf; **(K–M)** Detailed views corresponding to H–J; **(N)** ISIHL of RUBPC (RBCL) of longitudinal direction of leaf; **(O–Q)** ISIHL of PEPC at bottom, middle, and tip of leaf; **(R–T)** Detailed views corresponding to O–Q; **(U)** ISIHL of PEPC of longitudinal direction of leaf. For LS structure of leaf, the tip is on the right. BS, bundle sheath; E, epidermis; H, hypodermis; M, mesophyll; WS, water storage. Scale bar in A–C, G–J, N–Q, U is 100 µm; in D–F, K-M, R–T is 50 µm.

**Figure 7:**
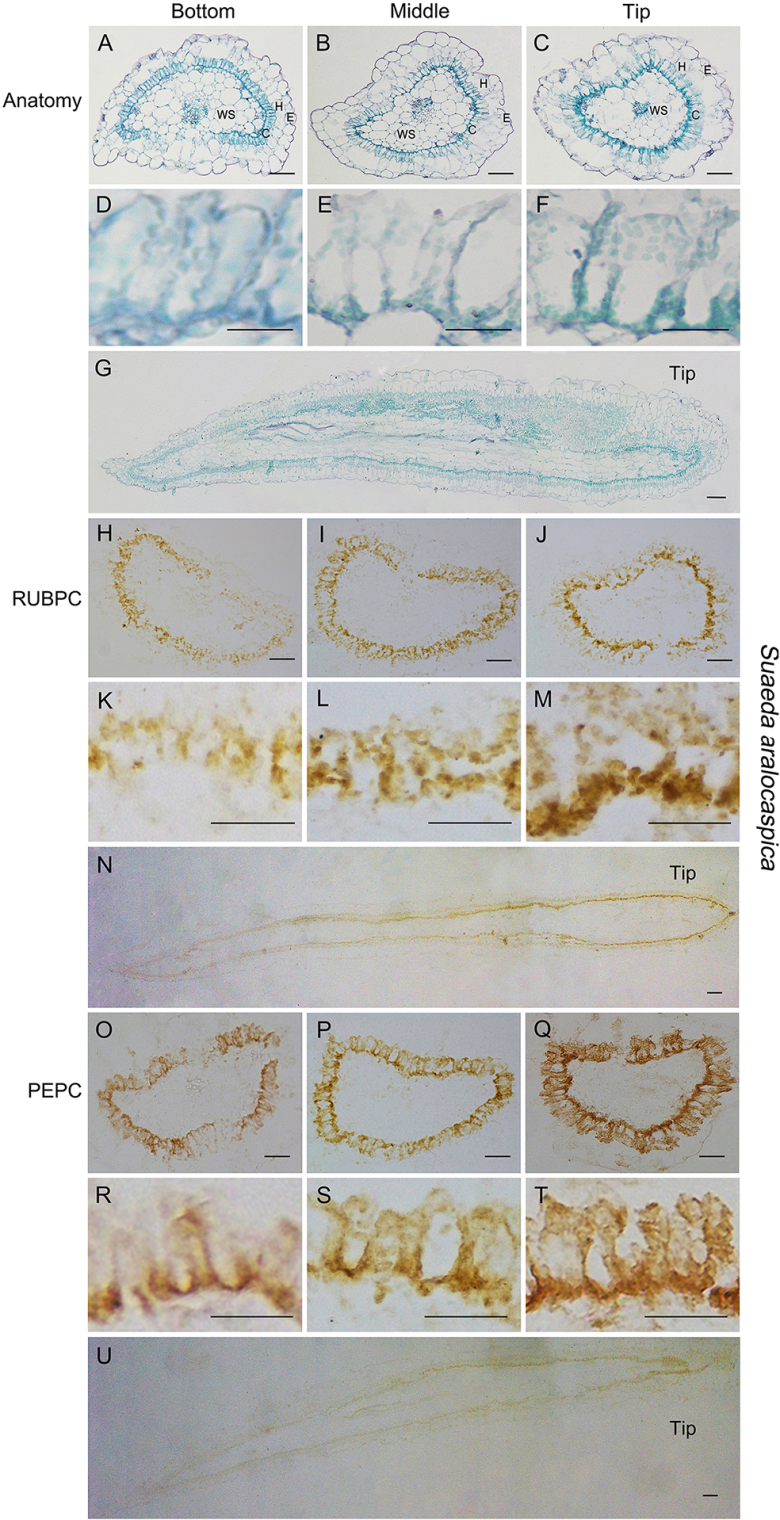
Anatomic structure of leaf and *in situ* immune-histochemical localization (ISIHL) of RUBPC and PEPC in *S. aralocaspica* with transverse and longitudinal sections of young leaves (0.5–0.6 cm), showing a progressive development with gradual structure differentiation. **(A–G)** Anatomic structure of young leaf; **(H–U)** ISIHL results; **(A–C)** Transverse sections from the bottom to tip of leaf; **(D–F)** Detailed views corresponding to A–C; **(G)** Longitudinal sections of leaf; **(H–J)** ISIHL of RUBPC (RBCL) at the bottom, middle, and tip of leaf; **(K–M)** Detailed views corresponding to H–J; **(N)** ISIHL of RUBPC (RBCL) of longitudinal direction of leaf; **(O–Q)** ISIHL of PEPC at the bottom, middle, and tip of leaf; **(R–T)** Detailed views corresponding to O–Q; **(U)** ISIHL of PEPC of longitudinal direction of leaf. For LS structure of leaf, the tip is on the right. C, chlorenchyma; E, epidermis; H, hypodermis; WS, water storage. Scale bar in A–C, G–J, N–Q, U is 100 µm; in K–M, R–T is 50 µm; D–F is 25 µm.

In both *S. ferganica* and *S. aralocaspica*, structural differentiation of BS and M cells (in *S. ferganica*) or chlorenchyma cells (in *S. aralocaspica*) of young leaves (0.5–0.6 cm long) was inspected using transverse (TS) and longitudinal (LS) sections under the microscope (**Figures 6** and **7**). *In situ* immuno-histochemical localization (ISIHL) was analyzed based on results of paraffin sections.

In *S. ferganica*, TS results showed that cells at the bottom of the leaf were less developed, BS cells presented as a discontinuous structure and were compact oblate in appearance, M cells were tightly arranged around the BS with thick cytosol and striking nucleus; while at the tip of the leaf, a continuous layer of BS cells was observed (**Figures 6A–F**). In addition, the cell volume became larger: M cells were enlarged and elongated, their cytosol became thinner, and chloroplasts became distributed along the radial cell periplasm. Compared to cells at the bottom, an interspace was introduced among M cells and between M cells and epidermal cells. LS results visualized a clear differentiation in BS and M cells from the bottom to the tip of the leaf, which presented a gradual developmental variation in structural specialization accompanying cell expansion (**Figure 6G**). Structure observations showed a higher differentiation in the tip cells while there was less among the bottom cells of the leaf. ISIHL analysis revealed that RUBPC was mainly distributed in BS cells and PEPC was widely distributed in the M cells in *S. ferganica* (**Figures 6H–U**). With photosynthetic tissue differentiated from the bottom to the tip of the leaf, the amount of RUBPC and PEPC protein was increased. Based on the analyses of TS and LS anatomic structure and enzyme localization we found that the expression of two enzymes was enhanced from the bottom to the tip of the leaf in *S. ferganica*.

In *S. aralocaspica*, TS and LS results showed that chlorenchyma cells near the leaf base were smaller and shorter, while cells at the tip were apparently developmentally advanced and similar to mature chlorenchyma cells, in which the chloroplasts were distributed to the proximal end or distal part, an apparent “chloroplast-free region” was also present in the middle part of chlorenchyma cells (**Figures 7A–G**). ISIHL analysis showed that the amount of RUBPC and PEPC protein increased with the leaf maturation, and from the bottom to the tip cells of the leaf (**Figures 7H–U**). The distribution of RUBPC was apparently concentrated to the proximal end in the tip cells compared to an irregular distribution in the bottom cells of young leaves (**Figures 7H–N**); PEPC was enhanced around the whole periplasm of chlorenchyma cells (**Figures 7O–U**).

**Figure 8:**
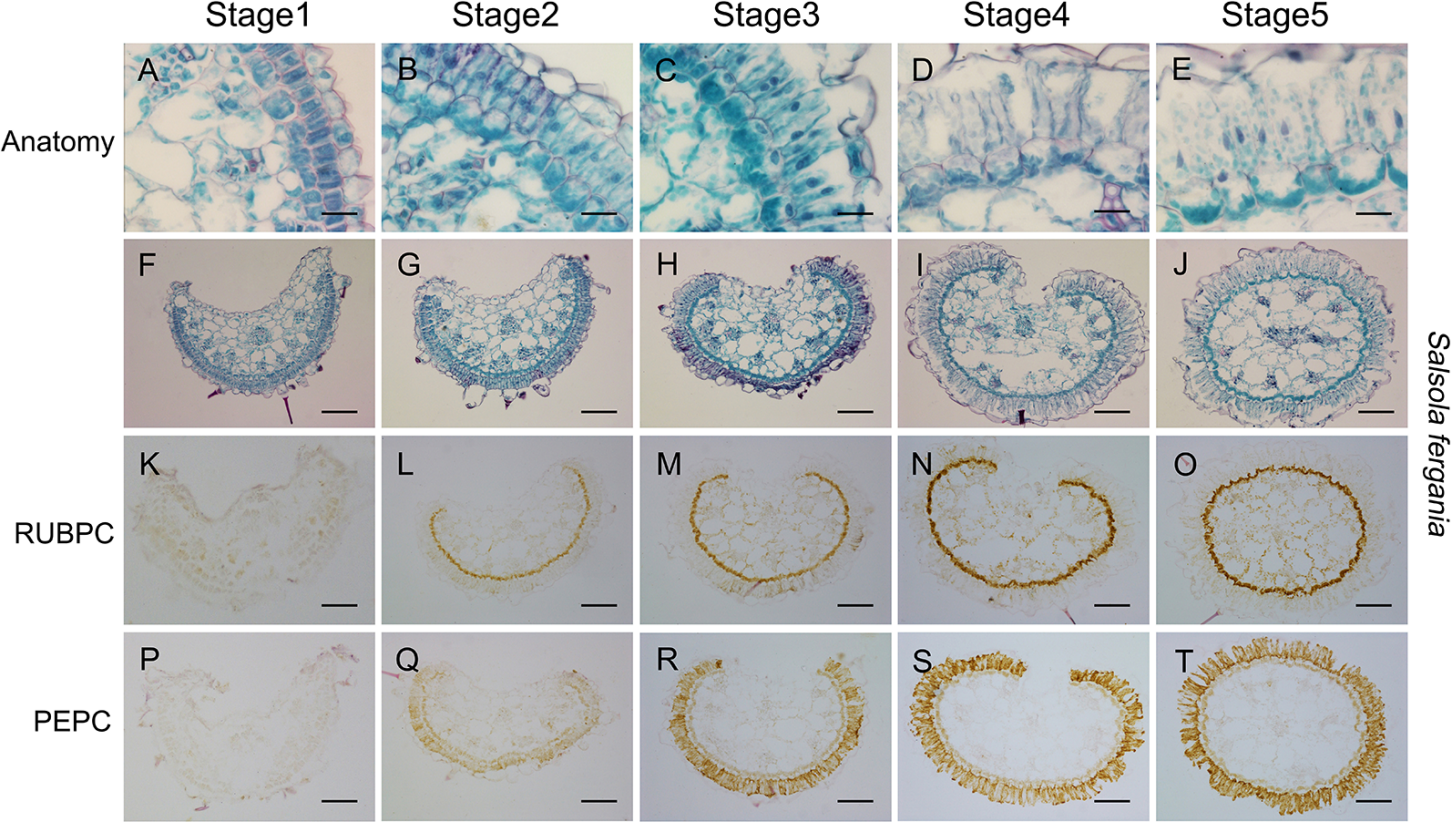
Anatomic structure of leaf and *in situ* immuno-histochemical localization (ISIHL) of RUBPC and PEPC of *S. ferganica* with transverse sections in the middle part of leaves at different developmental stages, showing a developmental gradient of structure differentiation. **(A–J)** Anatomic structure; **(K–T)** ISIHL results; **(A–E)** Middle part of transverse sections of leaf at different developmental stages (0.2 cm, 0.5 cm, 1.0 cm of leaf); **(F–J)** Detailed views of bundle sheath and mesophyll cells corresponding to images in A–E; **(K–O)** ISIHL of RUBPC (RBCL) corresponding to images in A–E; **(P–T)** ISIHL of PEPC corresponding to images in A–E. BS, bundle sheath; E, epidermis; H, hypodermis; M, mesophyll; VB, vascular bundle; WS, water storage. Scale bar in A–E is 25 µm; in F–T is 100 µm.

Apart from the longitudinal gradient development of young leave, photosynthetic structural and biochemical enhancement was also observed with plant development progression. In *S. ferganica*, clear differentiation in BS and M cells was present from early seedling (0.2 cm leaf) to mature seedling (1.0 cm leaf) (**Figures 8A–J**), both types of cells were expanded, the BS cells were arranged from discontinuous to continuous, and M cells were elongated. These changes presented a similar pattern with the differentiation in the same leaf from the bottom to the tip (**Figure 6**). ISIHL analysis showed that RUBPC was mainly distributed in BS cells while PEPC was in the whole periplasm of M cells (**Figures 8K–T**). With the maturation of the leaf, the expression of RUBPC and PEPC was increased.

**Figure 9:**
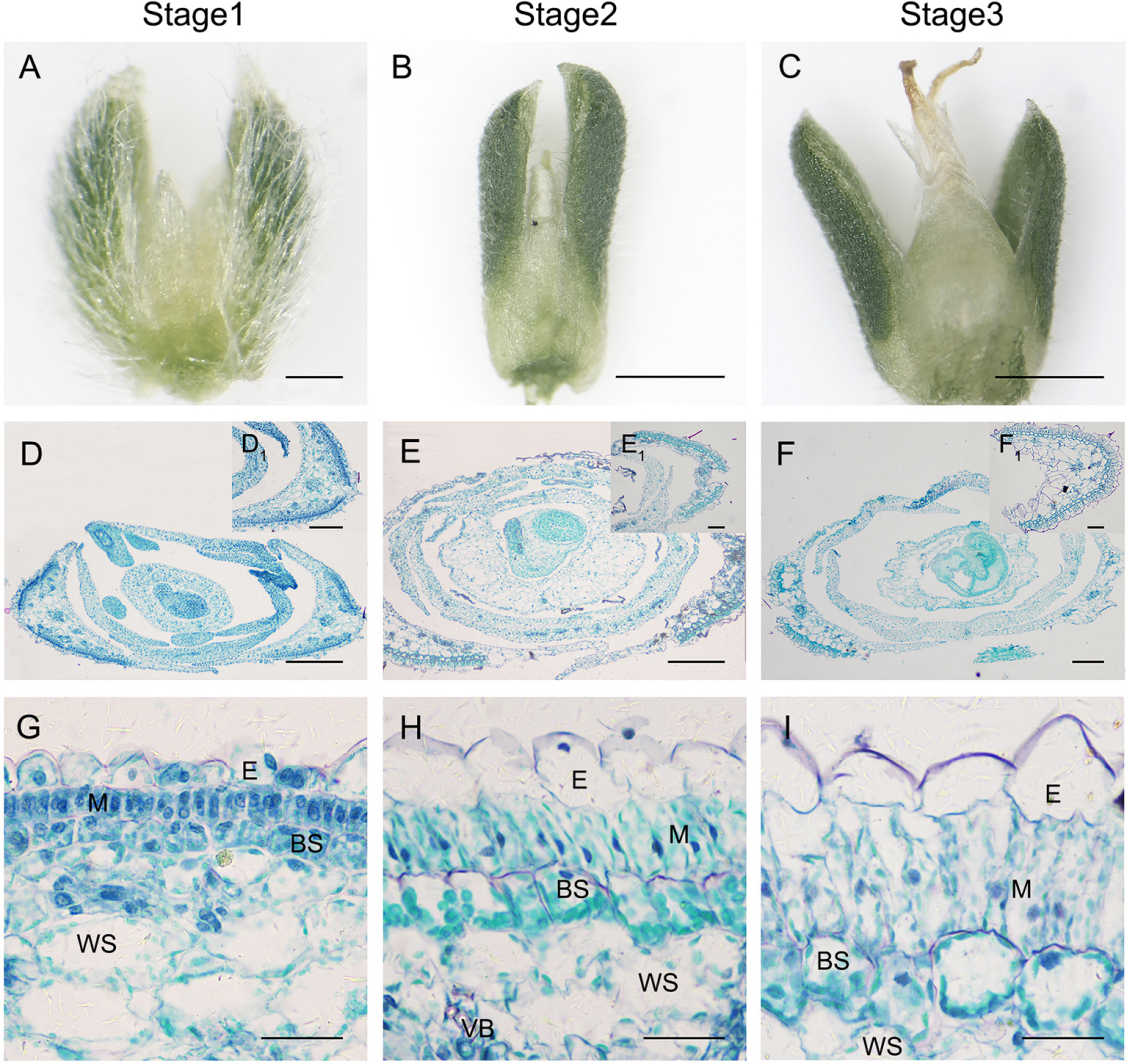
Anatomic structure of sepal in *S. ferganica* with transverse sections in the middle part of the flower. **(A–C)** Developing flowers, showing sepals on both sides; **(D–F)** Transverse sections of developing flowers; (D_1_–F_1_) Sepal section insets in the upper right position of D–F; **(G–I)** Detailed views of anatomic structure of sepal. Stage 1, Stage 2, Stage 3: different developmental times of flower. BS, bundle sheath; E, epidermis; H, hypodermis; M, mesophyll; VB, vascular bundle; WS, water storage. The images were acquired from the middle part of the flowers of indoor plant. Scale bar in A, (D_1_–F_1_) is 100 µm; in B, C is 500 µm; in D–F is 200 µm; in G-I is 50 µm.

Besides the above findings, we also found that photosynthetic structures in sepals also presented a differentiation gradient similar to that observed for C_4_ type Kranz-anatomy (**Figures 9A–I**), although the flower would produce an embryo with C_3_-type cotyledons. With flower development, both BS cells and M cells in sepals were expanded and extended, chloroplasts were differentiated and re-located around cells (**Figures 9G–I**).

### Transcriptional Expression Profiles of Photosynthetic Genes Along Longitudinal Leaf Direction in *S. ferganica* and *S. aralocaspica*

**Figure 10:**
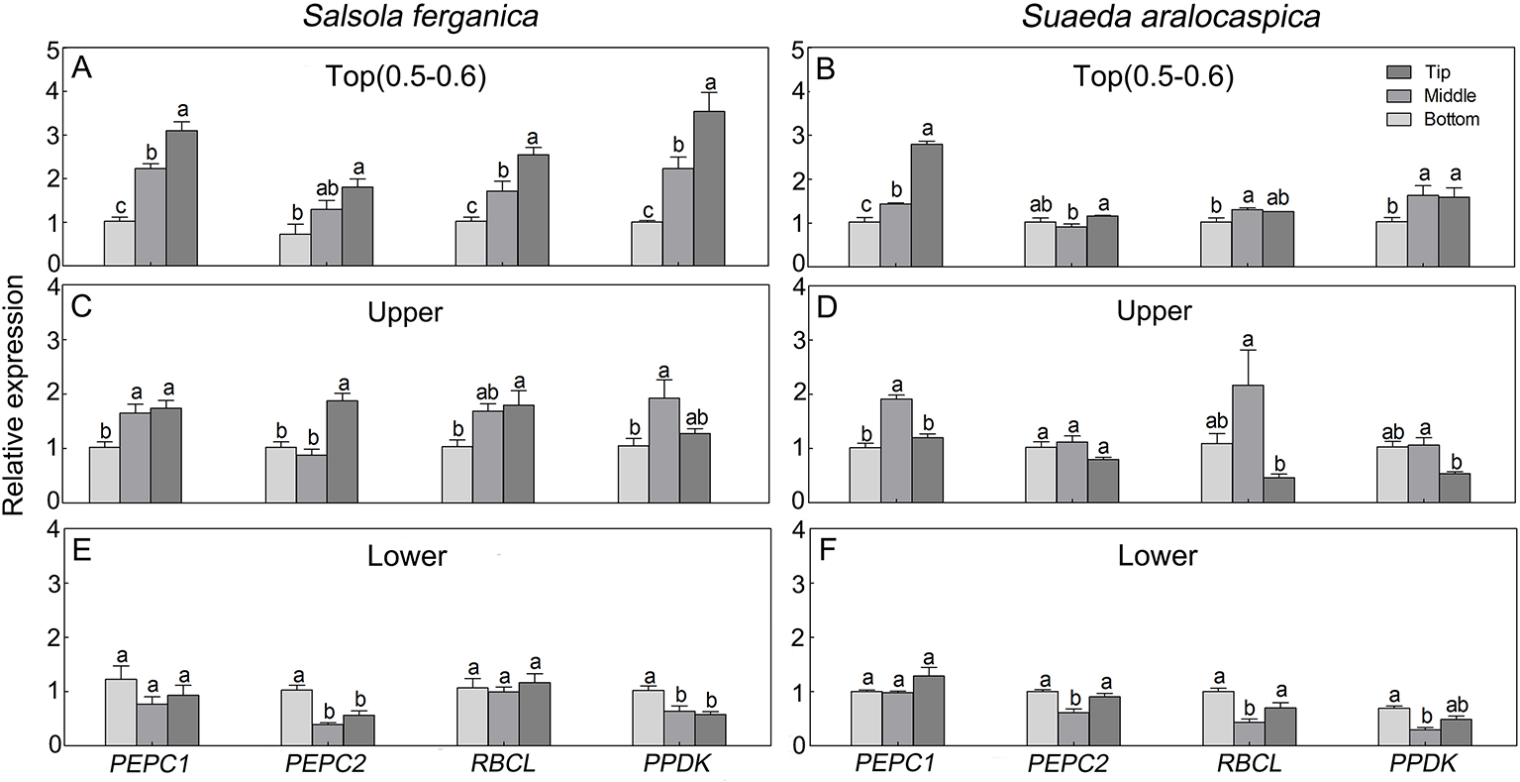
Transcriptional expression patterns of key photosynthetic genes in leaf of different positions of plant or along longitudinal direction in *S. ferganica* and *S. aralocaspica*. **(A, C, E)** *S. ferganica*; **(B, D, F)** *S. aralocaspica*; **(A, B)** 0.5–0.6 cm young leaf in top of plant; **(C, D)** Upper part leaf of plant (1.3–1.5 cm leaf in B; 1.8–1.9 cm in E); **(E, F)** Lower part leaf of plant (1 cm leaf in C; 2 cm in F). Bottom, Middle, Tip: Different segments along longitudinal leaf gradient. All samples were collected from indoor plants. Relative expression level is represented as the sample transcripts subtract the reference transcripts. Different lowercase letters above columns indicate signiﬁcant differences (*P* < 0.05 or 0.01) of the same gene between positions in one leaf. Values are means ± SE of three biological and two technical replicates (total of six replicates).

To investigate expression patterns of photosynthesis-related genes in leaf development in *S. ferganica* and *S. aralocaspica*, qPCR analyses of *PEPC*1, *PEPC*2, *PPDK*, and *RBCL* (large subunit gene of *RUBPC*) in leaves located in different positions of the plant or along the longitudinal direction of the leaf were conducted. Results showed that, for whole plants, the highest expression level was found in the top part (**Figures 10A, B**), the next was that of the upper part (**Figures 10C, D**), the lowest was in the lower part of leaves (**Figures 10E, F**); for along the longitudinal direction of a leaf, four genes were remarkably up-regulated from the bottom to the tip, especially in young leaves (0.5–0.6 cm) (**Figures 10A, B**). We also distinguished a transcriptional expression profile between *PEPC*1 and *PEPC*2, which we had also identified in our previous work. These two genes presented a similar expression pattern, in which *PEPC*1 was more active than *PEPC*2 in different parts of the plant or at different longitudinal positions of the leaf.

### Translational Expression Patterns of Photosynthetic Enzymes Along Longitudinal Leaf Direction in *S. ferganica*

**Figure 11:**
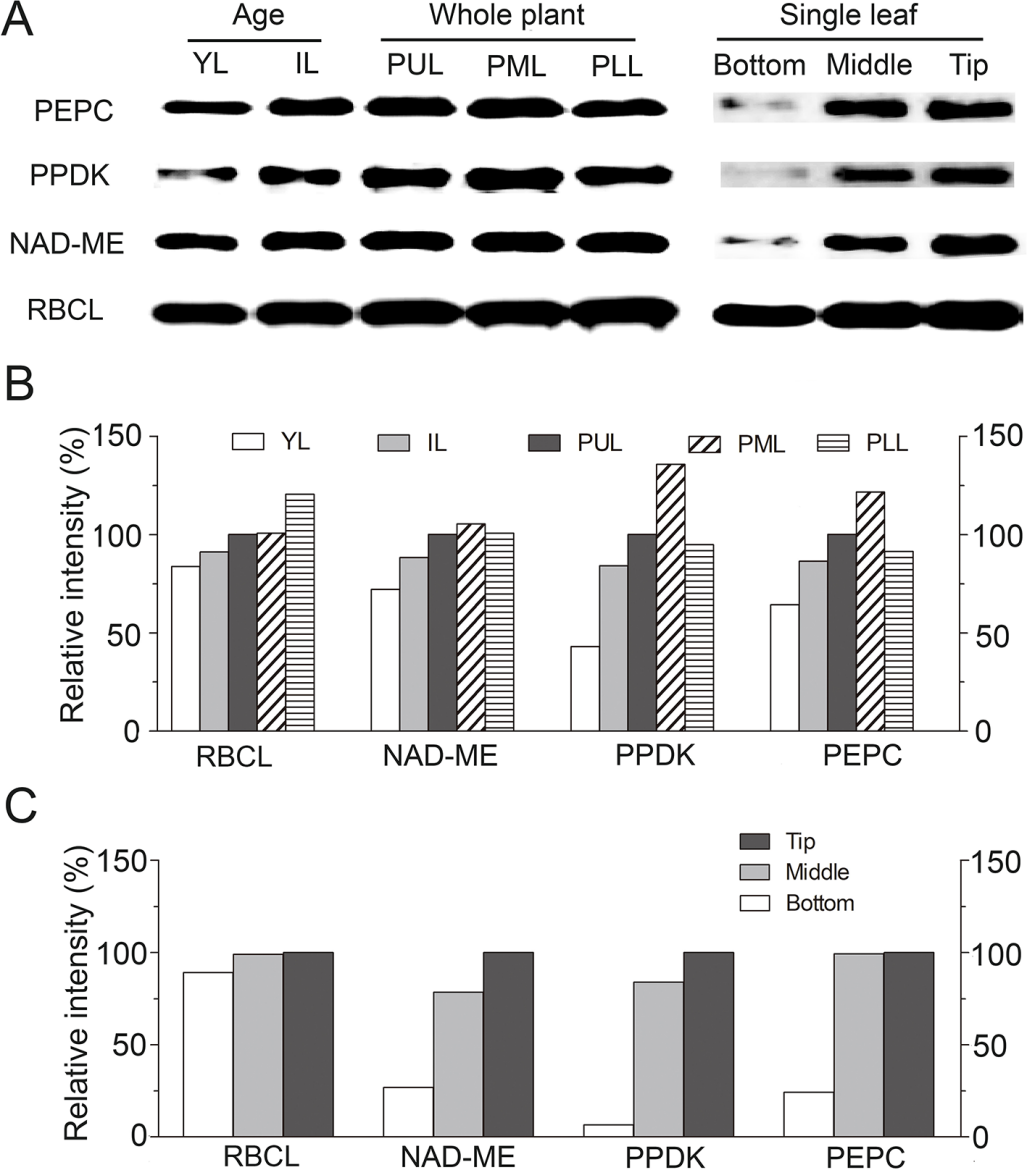
Translational expression patterns of key photosynthetic enzymes in leaf of different positions of plant or along longitudinal direction in *S. ferganica*. **(A)** Western blotting showing the detection of four proteins; **(B, C)** Quantitative expression of western blotting data by ImageJ. YL, young leaf (0.2–0.3 cm); IL, intermediate leaf (0.5–0.6 cm); PUL, PML, PLL: the upper, middle, lower layer of the plant; Bottom, Middle, Tip: Different segments, along longitudinal leaf gradient. Relative intensity (RI) is represented as the sample intensity divides the reference intensity. For RI of each enzyme in YL, IL, PUL, PML, and PLL, the ‘PUL’ was used while for bottom, middle, and tip of leaf, the 'Tip' was used as reference in calculation. All samples were collected from indoor plants. Total soluble proteins were extracted from different ages of leaves or different parts of single leaf. Protein loading amount in each lane was 10 μg. Preparation and dilution of first and secondary antibodies were described in Materials and Methods.

Translational accumulation of the representative C_4_ enzymes [RBCL (large subunit of RUBPC), NAD-ME, PPDK, PEPC] was analyzed by western blotting during leaf development in *S. ferganica* (**Figure 11**). All four proteins increased gradually as development progressed from the bottom to the tip of a single leaf, and with the highest level found in PUL or PML in mature leaves (**Figures 11A–C**). Among these, RBCL appeared to be the most abundant, this would be the expected given level of RBCL typically found in leaves (Koteyeva et al., 2011a; Koteyeva et al., 2014; Koteyeva et al., 2016), which increased along the gradient from the lower (PLL) to the middle (PML) or the upper (PUL) leaves of the plant. PEPC, NAD-ME, and PPDK were also remarkably accumulated as leaf development progressed and along the longitudinal direction of a single leaf. The bottom segment of young leaves presented with considerably lower levels of these proteins, except for RBCL. In total, the accumulation of the soluble protein of each enzyme showed initially a lower level in young leaves or the lower leaves of plants or at the bottom of a single leaf, and at higher levels in mature leaves or the upper leaves of plants or the tip of a single leaf.

## Discussion

*Salsola* is a large genus in Amaranthaceae, in which most species belong to halophytes or xerophytes and are distributed in extremely harsh environments (Akhani et al., 1997). A variety of structural and physiological adaptation mechanisms have been formed in these species in long-term evolutionary processes, among which the diversity of photosynthetic assimilation pathways is one of the typical characteristics in response to stresses (Pyankov et al., 2010). *S. ferganica* is one of the halophytes in *Salsola*, which possesses an enhanced ability for adaptation to adverse environments (Huang, 2005). So far, documentation suggests that *S. ferganica* has C_4_ Kranz-type structure and its δ^13^C value is in the range of C_4_ plants (Wen and Zhang, 2011). However, we found that large changes existed in leaf anatomic structures at different developmental stages and in some photosynthetic physiological behaviors in this species. Based on the previous findings, in the present study, we tried to explore the possible mechanisms of enhancement in photosynthetic structures and corresponding enzyme activities and gene expression at transcriptional and translational levels with plant development, as well as the behaviors of photosynthetic physiology in response to varying conditions in *S. ferganica*. Our results revealed that cotyledons had typical C_3_ structures in *S. ferganica*; while in leaves the photosynthetic structures, chloroplast dimorphism, PEPC:RUBPC ratio, starch staining, PEPC and RUBPC localization all presented as C_4_-Kranz Salsoloid type; moreover, with leaf or plant development, the anatomic structures and corresponding biochemistry of C_4_ syndrome were enhanced, i.e. in coordination with the changes of photosynthetic structures, the enzyme activity and gene expression (in transcriptional and translational levels) were increased from the lower to upper part of a plant and from the bottom to the tip of single young leaves. Besides, we also found that PEPC and RUBPC behaviors in *S. ferganica* were different from the typical C_4_ species *S. aralocaspica*. Whether our results indicate *S. ferganica* as being in a unique status during an evolutionary process for a photosynthetic pathway or not remains unknown; however, our findings should contribute to better understanding of diversity of C_4_ photosynthetic pathways in developmental and physiological aspects.

The prominent characteristics in determination of photosynthetic types are anatomic structures, which are usually associated with biochemical features (Ku et al., 1983). It has been documented that at least four major types of anatomic structures of C_4_ assimilation tissues exist in Chenopodiaceae, i.e. Atriplicoid, Kochioid, Salsoloid, and Suaedoid (Freitag and Stichler, 2000). In the present study, we visualized the significant difference in photosynthetic structure between cotyledon (C_3_ type) and true leaf (C_4_ type) in *S. ferganica*, the latter presented as continuous layers of BS and M cells surrounding WS cells and vascular tissues in well-developed leaf, which belongs to Salsoloid C_4_ type based on reported classification (Freitag and Stichler, 2000). In *Salsola*, most species have Salsoloid anatomy with typical Kranz BS cells and C_4_ assimilation pathway, e.g. *Salsola arbuscula*, *Salsola chivensis*, etc. (Freitag and Stichler, 2000; Voznesenskaya et al., 2001b). It has been proposed that Salsoloid anatomy evolved from Sympegmoid type (Akhani et al., 1997), the latter presents two-to-three layers of M cells, instead of Kranz cells, a discontinuous layer of BS cells is arranged adjacent to the peripheral bundles, e.g. *Salsola webbii* (C_3_), *S. arbusculiformis* (C_3_-C_4_) in *Salsola*, and C_3_ species in genus *Sympegma* (Voznesenskaya et al., 2001b). Besides anatomic structures, the ratio between MC and BSC area is also important for classification of C_4_ photosynthetic type (Bongard-Pierce et al., 1996). In *Salsola*, MC : BSC ratio in different assimilation types probably ranges around 9–15 (C_3_), 5–10 (C_3_-C_4_), or 2–5 (C_4_) (Voznesenskaya et al., 2013). In the present study, *S. ferganica* showed a ratio of 1.42, which fell into C_4_ category. C_4_ plants with Salsoloid-type Kranz anatomy, e.g. *Caroxylon orientale* and *Xylosalsola richteri*, present ratios of 4.5 and 1.9, respectively (Voznesenskaya et al., 2013). Generally, C_4_ structure is coupled with dimorphic chloroplast partitioning. Chloroplasts in BS cells are usually different in size, structure, function, etc., from those in M cells. In the classical C_4_ plant *Zea mays*, chloroplasts in BS cells are deficient in grana compared to M cells (Prakitchai et al., 2016); whereas in different Kranz-type species of Chenopodiaceae, MC chloroplasts have a reduced grana size and granal index (the length of all appressed thylakoid membranes as a percentage to total length of all thylakoid membranes in chloroplasts) compared to BSC (Gamalei and Voznesenskaya, 1986; Ueno, 2005). In SC-C_4_ species *B. sinuspersici*, dimorphic chloroplasts are biochemically located in the peripheral cytoplasm (PC, for C_4_ cycle) or central compartment (CC, for C_3_ cycle) around the nucleus: chloroplasts in PC are bigger than that of CC ones (Sascha et al., 2011). In the closely related SC-C_4_ species *B. cycloptera*, the granal index of CC chloroplasts is much higher than that in PC chloroplasts, which may suggest reduced Photosystem II activity in PC chloroplasts (Voznesenskaya et al., 2002). In the present study, we found that, in *S. ferganica*, larger, oval chloroplasts were distributed in BS cells, while smaller, long thin oval chloroplasts were located in M cells; in the SC-C_4_ plant *S. aralocaspica*, dimorphic chloroplasts were distributed in opposite ends of elongated chlorenchyma cells, larger ones were in the proximal ends and smaller ones in the distal ends in our experiment. However, the details of internal structure of dimorphic chloroplasts in the above two species need further experiments to clarify the extent of specialization between the types. Starch is presumably used as carbon and energy sources in leaf development. Previous studies showed that starch could be accumulated in all cell types of immature maize (classical C_4_ species) leaf tissue, but upon maturation, accumulation stops in mesophyll cells (Weise et al., 2011). In the present study, in the C_3_ type of cotyledon in *S. ferganica*, starch granules were randomly distributed in the mesophyll cells, while in mature C_4_ leaves starch was mainly distributed at the bottom of the BS cells; in *S. aralocaspica*, starch granules were mainly distributed in the periplasm of WS and hypodermal cells in incipient leaves, while in mature leaves, starch became accumulated at the proximal ends of chlorenchyma cells. Among different C_4_ species in Chenopodiaceae, only Rubisco-containing chloroplasts tend to accumulate starch (Voznesenskaya et al., 1999; Voznesenskaya et al., 2003; Voznesenskaya et al., 2005), which suggests that the presence of starch is closely associated to re-location of photosynthetic enzymes (Lara et al., 2008). Based on above data, we further analyzed and confirmed that RUBPC and PEPC of *S. ferganica* were localized in BS cells and M cells, respectively, while those of *S. aralocaspica* were distributed at the proximal end (RUBPC) and the whole chlorenchyma cell (PEPC), which was consistent with the previous report (Voznesenskaya et al., 2013). Taken together, the characteristics in anatomy, ratio of MC: BSC, dimorphic chloroplast differentiation, starch staining, and localization of photosynthetic enzymes suggest that *S. ferganica* belongs to a Salsoloid C_4_ photosynthetic type.

On the basis of anatomy, physiological behaviors, e.g. activities of photosynthetic enzymes, are also important in determination of carbon assimilation status for C_4_ species (Voznesenskaya et al., 2001b). In general, the activity of PEPC is about 50 times higher in C_4_ plants, and it is about 3 times higher in C_3_-C_4_ intermediate plants, than that of a typical C_3_ type (Voznesenskaya et al., 2001b). In the present study, PEPC activity (PA) in *S. ferganica* was lower than that of *S. aralocaspica*, especially in outdoor plants. In maize, PA in leaves presents the highest value at noon while 6 times or 3 times lower in the morning or in the afternoon (Peng et al., 1998). Other reports on maize indicate that under optimal conditions PA is higher and no diurnal change is observed; whereas under suboptimal conditions it is reduced by over 90% and presents a “unimodal” trend in response to light intensity variation (Kalt-Torres et al., 1987). In the present study, a similar expression pattern of PA diurnal variation was found in these two species exhibiting a “bimodal” curve, the lowest value was observed at 14:00 (2:00 pm) in the afternoon; however, the fluctuation of the PA values in *S. ferganica* was much smaller compared to that of *S. aralocaspica*. We also found that PA of *C. glaucum* and *N. tabacum* (C_3_ species) were insensitive to light intensity in the present study. It has been reported that in C_3_ plants PA has no significant change over a whole day; however, strong light and high temperature at noon enhance PA in C_4_ species like maize (Peng et al., 1998). Light regulation of PA has also been reported in other C_4_ species. Compared to maize, *Salsola soda* displayed a more substantial effect on PA in response to light diurnal fluctuation (Karabourniotis et al., 1985). Our results on PA for the two C_4_ desert plant species seemed to present a “midday depression of photosynthesis,” which may be in response to the strong light intensity and high temperature in the afternoon (**Table 1**). Other photosynthetic enzymes also present different trends between C_3_ and C_4_ plants, the amount of RUBPC in C_3_ is 3–6 times higher than in C_4_ species (Ku et al., 1979). In rice, the increase of RUBPC activity manifests as a “bimodal” pattern, in which the midday depression is in between two increases at 10:00 (maximum) and after 14:00 (slight rise) for the stomatal closing under high temperature (Weng et al., 1999). In the present study, RA in *N. tabacum* (C_3_) and *S. ferganica* (C_4_) was significantly higher than that of *S. aralocaspica*, especially that of indoor plants. High temperature can significantly decrease RA (Markus et al., 2006). It may be the effect of acquisition of oxygenase activity of RUBPC under rising temperature (Laing et al., 1974). Our results suggest that in *S. ferganica* PEPC and RUBPC behave as non-typical C_4_ species compared to that of SC-C_4_ species of *S. aralocaspica*.

In the present study, PA and the ratio of PEPC : RUBPC (P:R) in *S. ferganica* were lower (3–5 times lower in activity; 2–5 times lower in ratio) than that of *S. aralocaspica*, while they were remarkable higher than that of *C. glaucum* and *N. tabacum* (C_3_). The P:R ratio of C_4_ plants is usually larger while C_3_ species is less than 1, and C_3_-C_4_ intermediate in *Salsola* is generally less than 1 but higher than C_3_ species (Crespo et al., 1979). Our data showed that the P:R ratio in *S. ferganica* and *S. aralocaspica* was higher than 1, while that of the latter was much higher than the former. In addition, the performance of key photosynthetic enzymes between these two C_4_ species was apparently different. It is known that PA increase accompanying RA decrease with stress enhancement is a typical response in C_4_ species (Fontaine et al., 2003; Dizengremel et al., 2009). The increased PA can potentially improve carbon metabolism during a period of reduced stomatal conductance (Cushman and Borland, 2002; Carmosilva et al., 2008). Our results suggest that PA and RA in *S. ferganica* does not match with the typical C_4_ performance. Apart from enzyme activity, in the present study, another photosynthetic physiology index -δ^13^C in *S. ferganica* was also apparently affected by environmental variations, the value of outdoor (−16.15‰) or greenhouse (−21.73‰) plants presented much greater difference (*C. album* [C_3_]: −34.15‰, *N. tabacum* [C_3_]: −33.10‰ in greenhouse in our test), which was much higher than the previous reported value of −12.754‰ in *S. ferganica* (Wen and Zhang, 2011). The δ^13^C value is vulnerable to external environmental conditions such as water moisture, temperature, drought, etc. (Chen et al., 2002; Pan et al., 2016). Usually a difference of 4–7‰ is observed between indoor grown and outdoor grown plants (Voznesenskaya et al., 2013), which might explain our difference (5.58‰) between outdoor and indoor for *S. ferganica* plants, however, the value of the typical C_4_ plant *S. aralocaspica* was −14.87‰ in greenhouse which was much smaller than that of *S. ferganica* in greenhouse (−21.73‰) or outdoor (−16.15‰). Taken together, our results suggest that the key photosynthetic physiology in *S. ferganica* behaves with characteristics of a non-typical C_4_ species compared to SC-C_4_ type *S. aralocaspica*. Whether these differences arise because *S. ferganica* is at different evolutionary position in its development of its C_4_ photosynthetic pathway compared with *S. aralocaspica* remains to be a question for further exploration.

To complete C_4_ photosynthesis at high efficiency, a progressive development is usually coupled with leaf differentiation relating to photosynthetic structure and biochemistry in many C_4_ plant species (Koteyeva et al., 2016). Studies on a typical Kranz-anatomy species, *S. taxifolia*, revealed that a basipetally developmental mode of C_4_ structure and biochemistry is visualized by analysis of longitudinal leaf sections (Koteyeva et al., 2011a). In the present study, the anatomic structure and ISIHL analyses indicate that Kranz-anatomy C_4_ species *S. ferganica* presented a progressive development mode for both photosynthetic structure and biochemistry along the longitudinal gradient of young leaf, moreover, such gradient changes also applied to different developmental stages in leaves of lower, middle and upper parts of plants in coordination with the differentiation of BS and M cells, dimorphic chloroplasts, and enhancement of the photosynthetic enzymes’ expression and distribution. Our data suggest that, despite the independent origins and distinct photosynthetic structures, leaves of Kranz-type C_4_ species *S. ferganica* and SC-C_4_ species *S. aralocaspica* experienced similar base-to-tip transitions to form a C_4_ type in both structure and biochemistry. Such a phenomenon has been found in many different C_4_ species studied so far, including Kranz type, Kranz-like type, or SC-C_4_ species (Koteyeva et al., 2016). The representatives of Kranz-type C_4_ species from Poaceae have been reported to have M and BS cells that are differentiated along longitudinal gradients of leaf veins, accompanying with accumulation of enzymes or mRNAs for the C_4_ pathway (Langdale et al., 1988). In the C_4_ grass *A. hirta* (Poaceae), however, PEPC and RUBPC accumulation along the base-to-tip developmental gradient of leaves is not associated with veins (Wakayama et al., 2003). Different types of Kranz anatomy of C_4_ species in Chenopodiaceae (e.g. *S. taxifolia*, *S. eltonica*) and Cleomaceae (e.g. *C. angustifolia*) are differentiated basipetally and enhanced both in structure and biochemistry (Koteyeva et al., 2011a; Koteyeva et al., 2014). Besides developmental enhancement, in other C_4_ species, e.g. *S. aralocaspica*, at early developmental stage (i.e. seed germination), light can induce the transition of identical structure of plastids in the incipient chlorenchyma of cotyledons to form dimorphic chloroplasts, synthesize C_4_ enzymes, and generate structural and biochemical compartmentation, which ultimately leads to SC-C_4_ syndrome (Voznesenskaya et al., 2003). Such developmental or inducible enhancement of photosynthetic structure and biochemistry suggests that complete structural differentiation, in coordination with other C_4_ developmental processes, is essential for full C_4_ functions (Koteyeva et al., 2011a), which should have important biological and ecological significance in evolutionary processes and be a smart strategy in adaptation to harsh habitats.

A common feature of developmental enhancement of C_4_ syndrome is the differentiation of BS and M cells to form specialized functioning C_4_ photosynthetic tissues (Wakayama et al., 2003), therefore, the leaves located at different positions of the shoot may present distinct structure and biochemistry. For studying the progressive development of the C_4_ system, the leaf age (or size) and the distance to the leaf base are important (Koteyeva et al., 2011a). Leaf size less than 0.5–0.7 cm is suitable for observation of photosynthetic enhancement, while in fully expanded leaf (2–3 cm in *S. taxifolia*; 1.5–2 cm in *S. aralocaspica*) such phenomenon is not apparently present or has nearly disappeared (Koteyeva et al., 2011a; Koteyeva et al., 2016). In the present study, in *S*. *ferganica*, cell differentiation in distinct lengths (or ages) of leaves (e.g. 0.2 cm, 0.5 cm, 1.0 cm) and different positions on the shoot was diverse, 0.2 or 0.5 cm of leaf presented an enhanced development pattern along the longitudinal gradient; the differentiation degree in 0.2 cm leaf was relatively lower, in which the M cells were arranged neatly and compactly, and the BS cells were smaller, however, with the size increasing, the leaf around 1 cm was fully differentiated, both M and BS cells were significantly expanded, and BS cells were arranged from discontinuous to continuous, M cells were significantly elongated. So the leaf age (or size) is an important indicator for determination of developmental progression (Lara et al., 2008; Koteyeva et al., 2011a).

It has been suggested that biochemical compartmentation (e.g. PEPC accumulation in M cells) may serve as a developmental signal for structural differentiation (Dengler et al., 1995): such changes on photosynthetic enzymes should be regulated by relevant gene expression (Sheen, 1999). In classical C_4_ species (Kranz-anatomy), e.g. maize, corresponding to the progressive development of leaf structure, the majority of the photosynthetic genes are up-regulated in the tip of the leaf, which may be related to chloroplast differentiation or photosynthetic strategy (Cahoon et al., 2008). In SC-C_4_ species of *B. sinuspersici*, with the progressive transition from C_3_ mode to specialized functions of a C_4_ system, various photosynthetic genes are up-regulated significantly corresponding to the developmental enhancement in structure and biochemistry from the bottom to tip of the leaf (Lara et al., 2008). In the present study, we found that the transcripts of *RUBPC*, *PEPC*, and *PPDK* in *S. ferganica* or *S. aralocaspica* were accumulated from the lower to upper part of plant or the bottom to tip section of a young leaf, which was well-matched with the progressive enhancement of photosynthetic structure and biochemistry. In addition, we distinguished the expression pattern between *PEPC*1 and *PEPC*2 in *S. ferganica* or *S. aralocaspica* (Cheng et al., 2016), compared to the increase of *PEPC*1, *PEPC*2 altered in a limited range with the developmental enhancement of the C_4_ system. *PEPC*1 and *PEPC*2 encode two isoenzymes functioning in the phosphoenolpyruvate shuttle (O’Leary et al., 2011). It has been reported that *PEPC*1 regulates the carbon flux and lipid accumulation in the cell (Deng et al., 2014), while *PEPC*2 negatively affects intracellular lipid accumulation in *Chlamydomonas reinhardtii* (Deng et al., 2011). Previous work in *S. aralocaspica* revealed that two types of *PEPC* exhibited different expression patterns in response to various stresses (Cheng et al., 2016). In tomato (*Solanum lycopersicum*) fruit development, both *PEPC*1 and *PEPC*2 are regulated; however, under salt stress, the latter is up-regulated while the former gives no response (Yin et al., 2010). PEPC (1/2) from endosperm of castor seed is involved in fatty acid synthesis and the following malate production in leucoplasts (Blonde, 2003). So far, few studies have specialized on analyses between *PEPC*1 and *PEPC*2 and their impact on gradient development of photosynthetic structure. In maize, the tip-base ratio of *PEPC*1 transcripts in leaf is 68 times (Cahoon et al., 2008). In SC-C_4_ species of *B. sinuspersici*, a similar expression trend is observed (Lara et al., 2008). *PEPC1* and *PEPC2* perform different functions; however, which type contributes more in CO_2_ fixation still needs further evidence to clarify this point. Taken together, our data support the positive correlation between photosynthetic gene expression pattern and structural and biochemical enhancement of C_4_ syndrome in *S. ferganica* and *S. aralocaspica*.

The completion of the biochemical enhancement of C_4_ photosynthesis has to rely on the translational expression of relevant genes. A basipetal developmental progression of protein accumulation has been revealed in SC-C_4_ species of *B. sinuspersici* (Lara et al., 2008; Koteyeva et al., 2016), *B. cycloptera* (Voznesenskaya et al., 2005), and *S. aralocaspica* (Voznesenskaya et al., 2003; Koteyeva et al., 2016), in which Rubisco (rbcL) was accumulated earlier than other C_4_ enzymes in very young leaves, followed by a great increase of both PEPC and RUBPC activity in mature leaves (Koteyeva et al., 2014). In the present study, a gradient development in cellular differentiation and biochemical enhancement (e.g. rising expression of C_4_ photosynthetic enzymes) from the bottom to the tip of young leaves was also found in *S. ferganica* (Kranz anatomy). Analysis of the expression of proteins associated with C_4_ photosynthesis in *S. ferganica* showed that the related enzymes were large accumulated with the leaf progressive development both in different layers of the plant and along the leaf longitudinal gradient, in which a substantial RUBPC was present at the bottom of intermediate leaf (IL, 0.5–0.6 cm) during early development, meanwhile, a lag in the accumulation of C_4_ enzymes also existed, especially the PPDK, which might be a rate-limiting step for the developing C_4_ syndrome at an early stage (Lara et al., 2008; Koteyeva et al., 2011a; Koteyeva et al., 2014; Koteyeva et al., 2016). With the progression of structural differentiation and gene/protein expression, leaves located on different parts of the plant are driven to a more advanced developmental stage and proceed to a final mature C_4_ syndrome (Lara et al., 2008; Koteyeva et al., 2011a; Koteyeva et al., 2016).

## Conclusions

In the present study, we revealed the progressive development and enhancement of photosynthetic structure and biochemistry for C_4_ syndrome in a Kranz-anatomy species *S. ferganica*. Compared to SC-C_4_ species *S. aralocaspica* in our experiment*, S. ferganica* behaved as a non-typical C_4_ species in photosynthetic physiology, e.g. flexible δ^13^C value, lower PEPC activity, insensitive response to light intensity, etc. It is well-known that C_4_ plants are evolved for adaptation to harsh habitats (Su et al., 2012). The C_4_ assimilation pathway is a complicated system which has evolved across a long process and accumulated a variety of natural variations in anatomy and biochemistry related to the ancestral C_3_ forms (Sage et al., 2012; Heckmann et al., 2013; Covshoff et al., 2014), it means that different C_4_ types with different photosynthetic anatomy and physiology may survive diverse disasters in evolutionary processes. In the present study, on photosynthetic physiological characteristics, *S. aralocaspica* was stable while *S. ferganica* was more flexible in response to heterogeneous habitats: it has been suggested that the biochemical compartmentation in SC-C_4_ species is more advanced organization mode (Sage, 2002; Koteyeva et al., 2016). All these changes and differences among C_4_ species may represent different evolutionary steps for carbon assimilation pathways. Our findings revealed that both C_4_ species in this study shared similar developmental enhancement in their C_4_ system differentiation, and the completion of this system is essential for optimal practice of C_4_ photosynthesis (Voznesenskaya et al., 2003). Our results should contribute to further understanding of the C_4_ photosynthetic pathway in response to environmental variation. However, what the differences in photosynthetic physiology mean to C_4_ system evolution between these two C_4_ species needs more experimental evidence to elucidate.

## Data Availability Statement

All datasets generated for this study are included in the article/**Supplementary Material**.

## Author Contributions

HL, YL, and TM designed the experiments and methodology. YL and HL wrote the manuscript. YL, TM, and JZ conducted the experiments and collected the data. YL, TM, JZ, and YM analyzed the data. All authors contributed critically to the manuscript and gave final approval for publication.

## Funding

National Natural Science Foundation of China (31960037, 31660068); Open Funding of Key Laboratory of Xinjiang Uygur Autonomous Region (2016D03015).

## Conflict of Interest

The authors declare that the research was conducted in the absence of any commercial or financial relationships that could be construed as a potential conflict of interest.

## Supplementary Material

Supplementary Figure 1 Schematic diagram of location and position of cotyledon and true leaf of *S. ferganica* and *S. aralocaspica*. **(A)** *S. ferganica*; **(B)** *S. aralocaspica*. 0.5–0.6: 0.5–0.6 cm leaf; Upper: 1.3–1.5 cm leaf in A, 1.8–1.9 cm in B; Lower: 1.0 cm leaf in A, 2.0 cm in B. The image was taken from indoor plant. Scale bar is 1 cm.

Supplementary Figure 2 The developmental stage and morphology of *S. ferganica* in natural habitats. **(A)** Early seedling with two cotyledons only; **(B)** Seedling developed with small leaves covered with white, thick, long, and soft trichomes; **(C)** Plant with many branches and leaves covered with white, thick, long, and soft trichomes; **(D)** Plant initiates blossom covered with much thinner and shorter trichomes on leaf; **(E)** Plant at full blossom stage.
