# Supplementary figures and images for: The Developmental Enhancement of a C4 System With Non-Typical C4 Physiological Characteristics in Salsola ferganica (Kranz Anatomy), an Annual Desert Halophyte
Source: Front Plant Sci. 2020 Mar 6;11:152. doi: 10.3389/fpls.2020.00152 (PMC7069449; doi:10.3389/fpls.2020.00152)

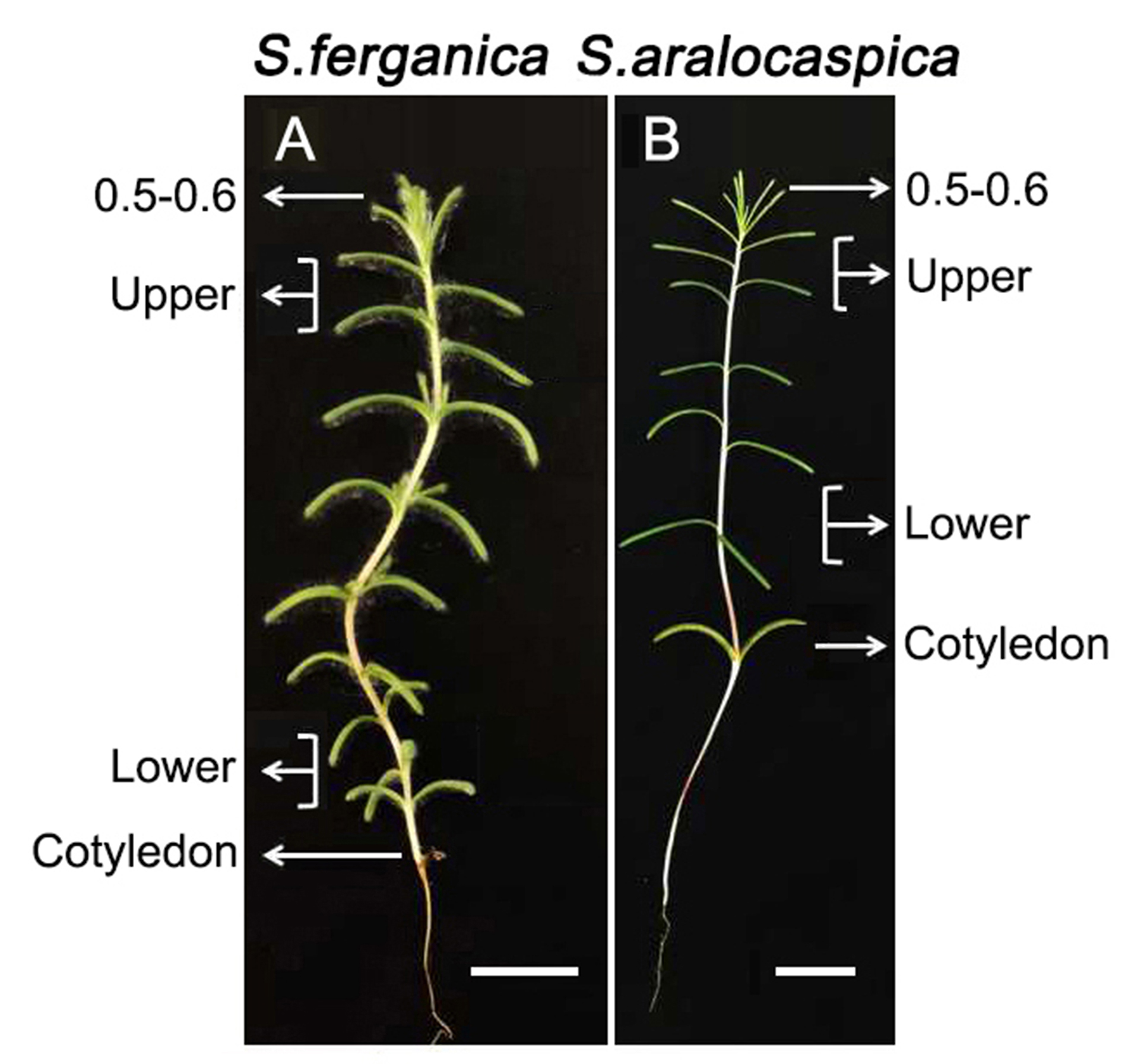

Supplement: Supplementary Figure 1 — Schematic diagram of location and position of cotyledon and true leaf of S. ferganica and S. aralocaspica. (A) S. ferganica; (B) S. aralocaspica. 0.5–0.6: 0.5–0.6 cm leaf; Upper: 1.3–1.5 cm leaf in A, 1.8–1.9 cm in B; Lower: 1.0 cm leaf in A, 2.0 cm in B. The image was taken from indoor plant. Scale bar is 1 cm. [file Image_1.jpeg]

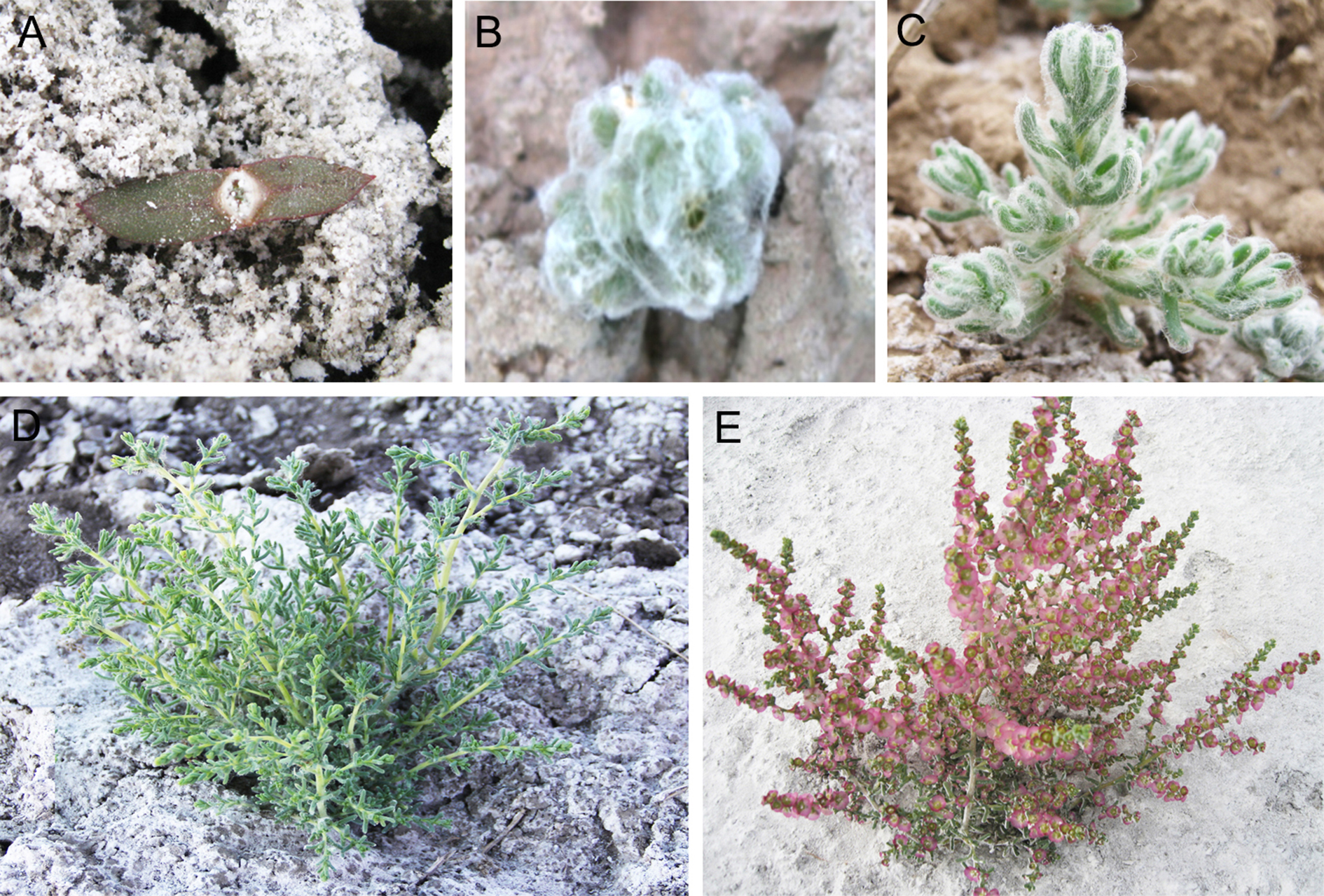

Supplement: Supplementary Figure 2 — The developmental stage and morphology of S. ferganica in natural habitats. (A) Early seedling with two cotyledons only; (B) Seedling developed with small leaves covered with white, thick, long, and soft trichomes; (C) Plant with many branches and leaves covered with white, thick, long, and soft trichomes; (D) Plant initiates blossom covered with much thinner and shorter trichomes on leaf; (E) Plant at full blossom stage. [file Image_2.jpeg]
